# Neonatal Sevoflurane Exposure Induces Long‐Term Cognitive Impairment via Epigenetically Mediated MMP9 Activation and Perineuronal Net Disruption in the Hippocampal CA2

**DOI:** 10.1002/advs.78006

**Published:** 2026-09-29

**Authors:** Lirong Liang, Jiachen Wang, Taozhi Wang, Yanling Li, Youyi Zhao, Guanghui Hao, Peiqin Gong, Ruling Chen, Yilin Fang, Shengxi Wu, Haopeng Zhang, Hui Zhang

**Affiliations:** ^1^ State Key Laboratory of Oral & Maxillofacial Reconstruction and Regeneration National Clinical Research Center for Oral Diseases Shaanxi Engineering Research Center for Dental Materials and Advanced Manufacture Department of Anesthesiology School of Stomatology Fourth Military Medical University Xi'an Shaanxi People's Republic of China; ^2^ Department of Anesthesiology Zhujiang Hospital Southern Medical University Guangzhou Guangdong People's Republic of China; ^3^ Department of Anesthesiology and Perioperative Medicine Xijing Hospital Fourth Military Medical University Xi'an People's Republic of China; ^4^ Department of Neurobiology and Institute of Neurosciences School of Basic Medicine Fourth Military Medical University Xi'an Shaanxi People's Republic of China

**Keywords:** H3K27me3, hippocampal CA2, matrix metalloproteinase 9, neonatal sevoflurane exposure, perineuronal nets

## Abstract

Sevoflurane is one of the most commonly used general anesthetics in pediatric clinical practice worldwide. Although accumulating preclinical evidence indicates that neonatal sevoflurane exposure causes persistent cognitive impairments, the extracellular mechanisms remain unclear. Herein, we focus on perineuronal nets (PNNs), extracellular matrix (ECM) structures that constrain neuronal excitatory plasticity and are highly enriched in the hippocampal CA2, a region critical for social recognition memory. Neonatal mice repeatedly exposed to 3% sevoflurane (2 h/day, P6‐P8) exhibited persistent CA2‐specific PNN degradation in adulthood. Mechanistically, sevoflurane depleted H3K27me3 at the *Mmp9* promoter, elevating matrix metalloproteinase 9 (MMP9) expression. This epigenetic dysregulation impaired BDNF/ TrkB signaling, reduced PSD‐95 puncta density and dendritic spine abundance, and suppressed mEPSC frequency, leading to impaired novel object recognition and social discrimination. Spatial transcriptomics validated CA2‐specific ECM pathway dysregulation. Notably, CA2‐targeted Hapln1 overexpression in the CA2 excitatory neurons of Camk2a‐Cre mice effectively restored PNN integrity, rescued synaptic dysfunction, and reversed cognitive deficits. The pharmacological inhibition of MMP9 yielded comparable neuroprotective effects. Collectively, this study identified PNNs as pivotal mediators of anesthetic neurotoxicity, uncovered a previously unrecognized epigenetic‐ECM coupling mechanism driving developmental brain injury, and highlighted PNN preservation as a promising translational strategy for preventing pediatric anesthesia‐associated cognitive impairment.

## Graphical abstract

Hippocampal CA2 perineuronal nets ensheathing excitatory pyramidal neurons are selectively vulnerable to repeated neonatal sevoflurane exposure. Epigenetic upregulation of MMP9 via reduced H3K27me3 disrupts PNN integrity, impairs BDNF–TrkB signaling, and causes synaptic protein loss and dendritic spine deficits, leading to persistent social and spatial cognitive impairment. Both pharmacological MMP9 inhibition and genetic Hapln1 restoration reverse these deficits.

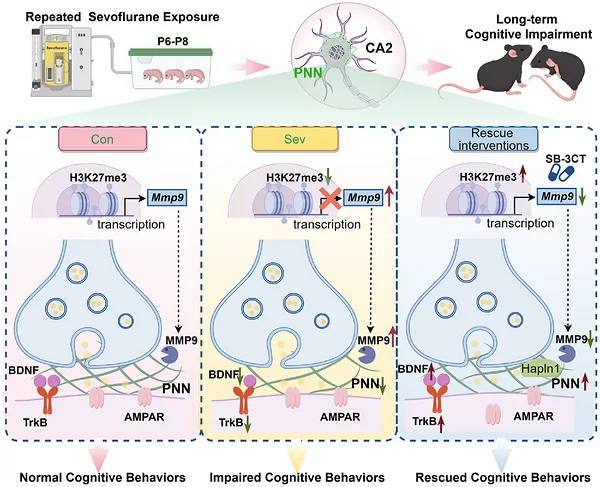

## 1 Introduction

Sevoflurane is the most widely used anesthetic in pediatric surgery. While its pharmacokinetic profile in adults is well characterized, its impact on the developing brain has emerged as a critical safety concern. Robust preclinical evidence has consistently associated neonatal exposure with persistent cognitive and social behavioral deficits [1, 2, 3], whereas clinical studies have yielded inconsistent results [4]. These findings have catalyzed intensive investigation into the mechanisms underlying developmental anesthetic neurotoxicity, revealing mitochondrial dysfunction, oxidative stress, aberrant tau phosphorylation, and synaptic dysregulation as key pathological features [5, 6, 7, 8, 9]. More recent studies implicate microglial synaptic pruning via complement pathways, pyroptosis, and gut‐brain axis alterations [10, 11, 12]. However, the field lacks a unified framework for the primary molecular triggers and persistent cellular alterations, and no mechanism‐based interventions have reached clinical translation.

The extracellular matrix (ECM) remains conspicuously overlooked in this field of research. While intracellular signaling and neuron‐glia crosstalk have dominated mechanistic inquiries [5, 13], the ECM, a fundamental regulator of synaptic stability and neural microenvironment homeostasis, has received scant attention [14, 15]. This is particularly consequential for perineuronal nets (PNNs), specialized chondroitin sulfate‐rich lattice structures that ensheath discrete neuronal populations, including parvalbumin‐positive interneurons and excitatory neurons in the hippocampal CA2 region [16, 17]. By stabilizing synaptic contacts and constraining plasticity after critical periods, PNNs serve as essential scaffolds for maintaining circuit fidelity in the mature brain [18]. PNN deterioration is causally associated with cognitive dysfunction in patients with multiple neurological pathologies [19]. Nevertheless, it remains unclear whether PNN degradation contributes to persistent cognitive deficits following neonatal sevoflurane exposure [20].

Structurally, PNN integrity is sustained by a tightly regulated equilibrium between ECM biosynthesis and proteolytic degradation [16]. Matrix metalloproteinase 9 (MMP9) is the principal enzyme driving PNN breakdown, directly cleaving the core constituents and dismantling the pericellular lattice [21]. MMP9 transcription is tightly regulated by epigenetic mechanisms. Trimethylation of lysine 27 on histone H3 (H3K27me3), a well‐characterized repressive histone mark, is enriched at the MMP9 promoter and acts as a key upstream transcription inhibitor [22]. This aligns with the broader evidence linking hippocampal H3K27me3 remodeling to memory‐related pathology, as observed in toxic exposure models of neurodevelopmental injury [23]. Notably, sevoflurane upregulates MMP9 expression and activity in developing neurons [24], suggesting an untested mechanistic pathway: sevoflurane‐induced reduction of H3K27me3 may de‐repress MMP9 transcription, driving excessive proteolytic cleavage of core PNN components. In the developing brain, excessive MMP9 activation disrupts ECM homeostasis and causes progressive PNN erosion [25]. Therefore, we propose an H3K27me3–MMP9–PNN regulatory axis, in which epigenetic dysregulation of MMP9 acts as an upstream initiating event that tips the ECM balance toward net degradation. While MMP9 contributes to PNN degradation, Hapln1 acts as a molecular guardian. This link protein cross‐links aggrecan to hyaluronan, reinforcing the PNN meshwork and protecting it from proteolytic dissolution [26, 27]. Reduced Hapln1 expression destabilizes PNN assembly, leading to aberrant synaptic plasticity, a vulnerability that we propose is exploited by neonatal sevoflurane exposure.

PNN integrity is functionally intertwined with brain‐derived neurotrophic factor (BDNF)/tropomyosin receptor kinase B (TrkB) signaling [28]. This pathway drives synaptic vesicle recycling, postsynaptic density (PSD) formation, and long‐term potentiation [29], whereas PNNs reciprocally regulate it by restricting BDNF diffusion and controlling TrkB localization at synaptic sites [28]. This creates a double‐lock system in which PNNs stabilize synapses by constraining BDNF availability, whereas BDNF signaling maintains the molecular machinery that PNNs protect. We hypothesize that sevoflurane breaks this lock—Hapln1 downregulation compromises PNN integrity, triggering aberrant BDNF diffusion and disrupting TrkB‐dependent synaptic maintenance, ultimately culminating in persistent cognitive deficits.

Here, we tested the hypothesis that neonatal sevoflurane exposure disrupts the PNN–BDNF/TrkB axis via the upstream H3K27me3–MMP9 pathway, thereby impairing synaptic stability and cognitive function. By positioning epigenetic‐mediated ECM dysregulation as a primary driver rather than a secondary consequence of anesthetic neurotoxicity, this work reframes the mechanistic landscape of developmental anesthetic injury and identifies tractable candidate epigenetic and ECM targets for further preclinical and translational investigation.

## 2 Results

### 2.1 Spatial Transcriptomics Reveals Distinct Sevoflurane‐Induced Hippocampal Gene Expression Signatures

Developmental exposure to general anesthesia impairs brain function in adulthood, affecting social interactions and cognitive abilities (Figure S1A–K). To identify the underlying molecular mechanisms, we employed spatial transcriptomics to profile gene expression in coronal brain slices obtained from sevoflurane (Sev)‐treated or control (Con) mice following 3 consecutive days of Sev exposure. We first characterized region‐enriched expression patterns across samples from both Con and Sev‐treated conditions (Figure 1A–D and Figure S2). Each coronal section covered between 3133 and 3760 spots on the Visium slides (Figure S2).

**FIGURE 1:**
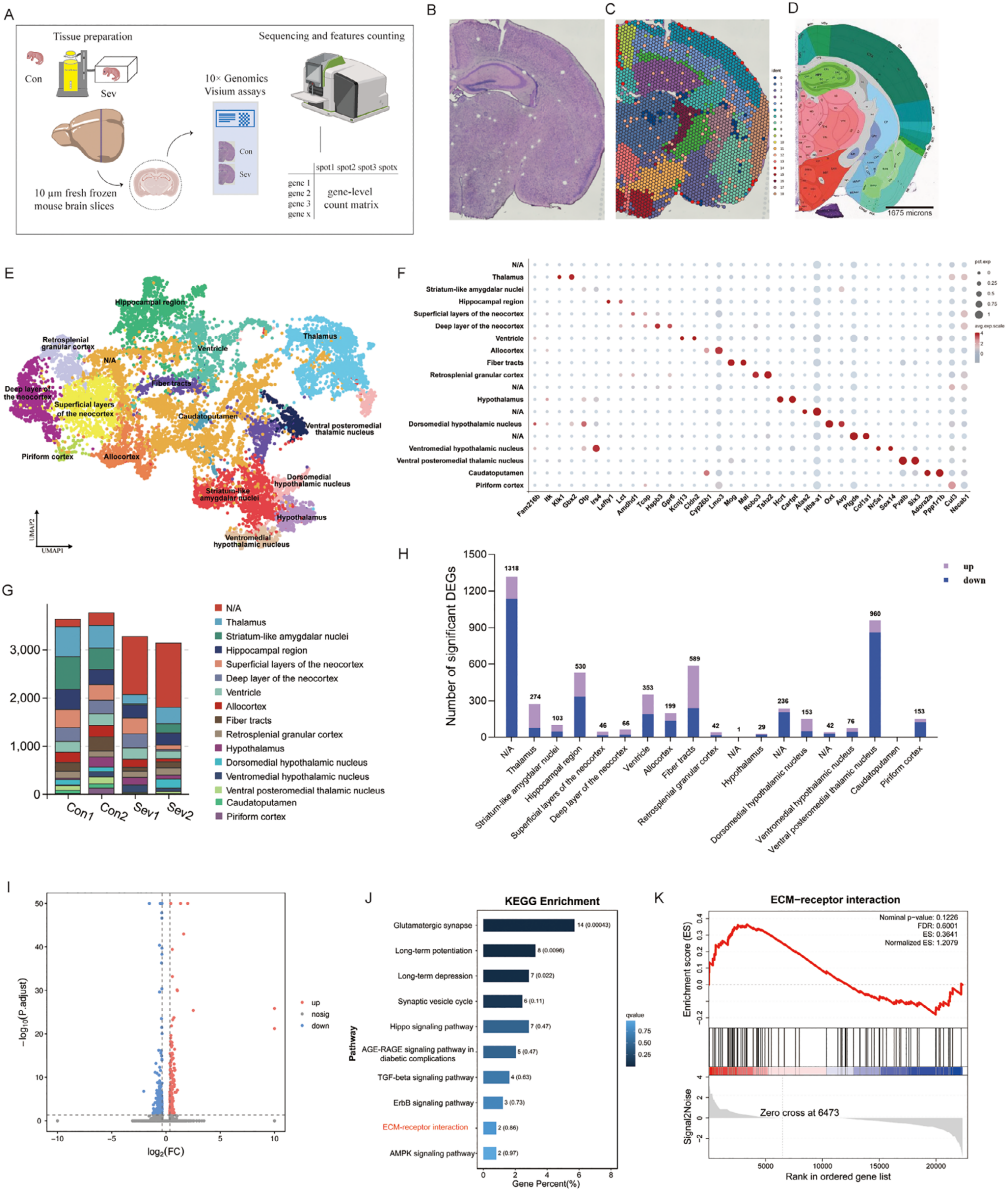
Anatomically discrete brain regions exhibit distinct spatial patterns of gene expression. (A) Schematic of Visium spatial transcriptomics workflows comparing sevoflurane (Sev) and control (Con) conditions. (B) Representative H&E‐stained coronal brain tissue section used for spatial transcriptomics. (C) Graph‐based clustering analysis of spot‐level data (3760 spots) from Sample 2. Each spot is color‐coded according to the transcriptional signature derived from 36 principal components via the Louvain clustering algorithm. Brain regions are annotated in the legend. (D) Adapted image from the Allen Mouse Brain Reference Atlas (coronal section, image 69/132, position 273; http://atlas.brain‐map.org/). (E) UMAP plot based on transcriptional signatures of individual spots. (F) Bubble chart of the most significant biomarkers for each brain region: bubbles denote biomarker expression levels across regions, and bubble diameter correlates with the proportion of spots expressing the biomarker. Two key significant biomarkers are displayed for each brain region. (G) UMAP plot depicting relative comparisons of spatial clusters across the entire sample set. (H) Bubble plot showing top significant biomarkers per brain region. Each bubble depicts a biomarker's expression level in a specific region, with size proportional to the percentage of spots expressing the biomarker. Two major significant biomarkers are included for each brain region. (I) Volcano plot showing differentially expressed genes (DEGs) in the hippocampus between Con and Sev groups. Upregulated DEGs are marked in red, downregulated ones in blue, with grey indicating non‐significant genes. (J) KEGG pathway enrichment analysis results of DEGs between Con and Sev groups. (K) Gene set enrichment analysis (GSEA) comparing Con and Sev hippocampal samples. *n* = 2 mice per group.

Predictably, this method identified brain regions with high accuracy (Figure 1C) and aligned with the anatomical labeling of the Allen Mouse Brain Reference Atlas [30] (Figure 1D). Each brain region exhibited distinct transcriptional signatures. Unsupervised clustering of region‐specific expression profiles identified well‐separated clusters (Figure 1E) and their representative top biomarkers (Figure 1F). Next, we examined the distribution of annotated brain regions across all four samples (Con1, Con2, Sev1, and Sev2). Spatial clustering patterns were highly consistent between the Con and Sev‐treated groups (Figure 1G). However, how Sev exposure alters transcriptomic activity in distinct brain regions remains unknown, because bulk sequencing methods inevitably average out region‐specific effects and mask the unique molecular signatures of each area. To fill this gap, we performed differential gene expression (DEG) analyses for each anatomically annotated brain region in our coronal spatial transcriptomic sections. Quantification of significant DEGs across all brain regions revealed striking heterogeneity in transcriptional responses to Sev exposure (Figure 1H). The number of DEGs varied widely across regions, with the unannotated region (N/A) harboring the most extensive transcriptional changes (1318 DEGs), followed by the Ventral posteromedial thalamic nucleus (960 DEGs) and hippocampal region (1318 DEGs). Notably, most brain regions had predominantly downregulated genes following Sev exposure, suggesting a widespread transcriptional repression effect.

Given the well‐established role of the hippocampus in learning and memory—functions known to be impaired by developmental Sev exposure—we next focused our mechanistic investigations on changes in hippocampal gene expression. Differential analysis of the hippocampal region revealed a distinct set of Sev‐responsive genes that were visualized using a volcano plot to highlight the significantly upregulated and downregulated transcripts (Figure 1I). Subsequently, Kyoto Encyclopedia of Genes and Genomes (KEGG) pathway enrichment analysis of these hippocampal DEGs identified critical biological processes perturbed by Sev, including glutamatergic synaptic transmission, long‐term potentiation, and ECM‐receptor interaction (Figure 1J). Gene set enrichment analysis (GSEA) further validated marked enrichment of the ‘ECM‐receptor interaction’ pathway in Sev‐exposed hippocampal samples, suggesting a central role for ECM remodeling in Sev‐induced neurotoxicity (Figure 1K). These spatially resolved transcriptomic data pinpointed specific hippocampal subfields as the primary sites of Sev‐induced ECM remodeling, and directly informed the design and direction of all subsequent mechanistic validation experiments.

### 2.2 PNNs in the CA2 Region of C57BL/6J Mice are Diminished Following Repeated Neonatal Exposure to Sevoflurane

To date, most mechanistic investigations of the effect of general anesthetics on cognition and behavioral outcomes have focused primarily on intracellular homeostatic regulatory mechanisms [5, 31]. In contrast, the functional contributions of the ECM, a key component of the neural microenvironment, have long remained underappreciated and poorly explored in the context of anesthetic‐induced neurotoxicity. In addition, providing structural support to neurons, PNNs actively regulate synaptic transmission and neural plasticity. These regulatory functions are essential for sustaining normal neural activity and intact cognitive behaviors [32]. The experimental timeline, outlining the schedule of neonatal Sev exposure and subsequent tissue collection for all analyses, is shown in Figure 2A.

**FIGURE 2:**
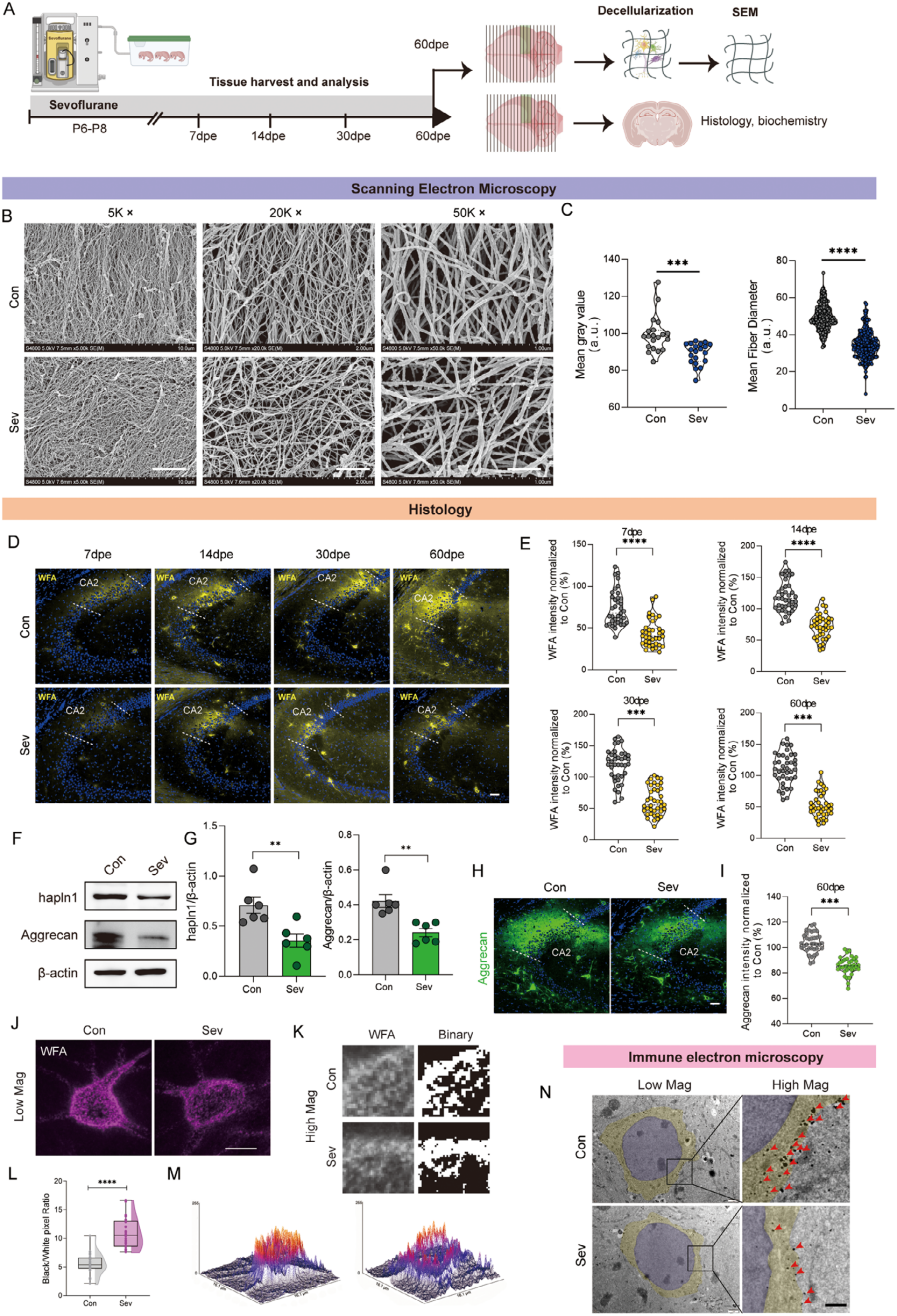
Neonatal sevoflurane exposure induces PNNs damage in the hippocampus. (A) Schematic of the experimental protocol for hippocampal extracellular matrix (ECM) analyses. (B) Representative scanning electron micrographs (SEM) of hippocampal ECM ultrastructure in Con and Sev ‑treated groups. Scale bars = 5 µm (5K×), 1.2 µm (20K×), and 500 nm (50K×). (C) Quantification of mean gray value (left) and fiber diameter (right) of hippocampal ECM in Con and Sev groups. (D, E) Wisteria floribunda agglutinin (WFA) staining and quantitative analysis of PNN intensity in the CA2 region at 7, 14, 30, and 60 days post‐exposure (dpe). Scale bar = 50 µm. (F, G) Western blotting and quantification of hapln1 and aggrecan protein levels in the hippocampus of Con and Sev groups. (H, I) Immunofluorescence staining and quantitative analysis of aggrecan in the hippocampus of Con and Sev groups at 60 dpe. Green: aggrecan. Blue: DAPI. Scale bar = 50 µm. (J–M) PNNs in the Con group exhibit bright, well‐organized mesh‐like architecture, whereas PNNs in the Sev group display reduced WFA intensity and a disrupted staining pattern. (J) Representative WFA staining. Purple: WFA. Scale bar = 10 µm. (K) Binary images of the regions in (J). (L) Quantification of black/white pixel ratio. (M) Representative surface intensity profiles of WFA‐positive PNNs. (N) Immunoelectron microscopy of PNNs in the Con and Sev groups. High‐magnification images (right panels) display immunogold labeling within WFA (red arrows) in both groups. Scale bars = 1 µm (left), 500 nm (right). Data represent the mean ± SEM. *n* = 3 mice per group for SEM (B, C, J–N); *n* = 6 mice per group for all other experiments. Statistical comparisons were conducted using unpaired Student's t‐test. ∗∗*p* < 0.01, ∗∗∗*p* < 0.001, ∗∗∗∗*P*< 0.0001.

To test the hypothesis that neonatal Sev exposure triggers widespread ECM remodeling within the adult hippocampus, we isolated hippocampal tissues from Con and Sev‐treated mice, followed by decellularization using a well‐established protocol to remove cellular components while preserving ECM integrity (Figure S3). Scanning electron microscopy (SEM) was used to characterize structural changes in the purified ECM. Compared with Con mice, Sev exposure disrupted the microarchitecture of hippocampal ECM. Specifically, the Sev group displayed sparser and thinner ECM fibers along with prominent alterations in structural geometric patterns (Figure 2B,C). To further explore how PNNs respond to repeated neonatal Sev exposure, we examined PNN expression in the hippocampus of Con‐ and Sev‐treated mice by staining with the classic PNN marker Wisteria floribunda agglutinin (WFA) [33]; hippocampal PNN intensity progressively declined at 7, 14, 30, and 60 days post‐exposure (dpe), indicating persistent anesthesia‐induced damage to the PNN structure (Figure 2D,E). Further regional analysis localized this PNN loss predominantly to the CA2 subfield. To assess whether the effect was CA2‐selective or extended to other hippocampal regions, we quantified WFA fluorescence intensity in the CA1, CA3, and dentate gyrus (DG) at 60 dpe. In contrast to the significant reduction observed in the CA2, WFA levels in the CA1, CA3 and DG were comparable between control and sevoflurane‐exposed mice, with no statistically significant differences detected (Figure S4A–C). These data indicate that sevoflurane‐induced PNN degradation is region‐specific, being largely restricted to the CA2. We further quantified the expression of core proteoglycans that constitute the hippocampal PNNs, which showed markedly decreased both Hapln1 and Aggrecan levels following Sev exposure (Figures 2F–I and Figure S4D–G).

To further quantify the morphological disruption of PNNs, we analyzed their fluorescence intensity and spatial distribution along the neuronal surface using binary gap analysis. This approach converts grayscale images into a binary black‐and‐white format based on an unbiased threshold calculation, enabling the precise evaluation of interstitial gaps within PNN structures. In the Con samples, binary imaging revealed clearly demarcated gaps and well‐organized lattice architecture of intact PNNs. In contrast, PNNs from Sev‐exposed mice exhibited blurred gap boundaries along with aggregated and discontinuous staining patterns that indicated large PNN‐absent regions (Figure 2J–L). Additionally, we assessed the sharpness of the PNN signal peaks on neuronal surfaces using the local maxima function in ImageJ (Figure 2M). Immunoelectron microscopy further confirmed compromised the ultrastructural integrity of WFA‐labeled PNNs in Sev‐exposed mice, accompanied by markedly reduced in immunogold particle density (Figure 2N). Collectively, these results demonstrate that Sev exposure induces severe structural impairment of PNNs in the hippocampal CA2 region. This disruption of PNN structure, along with the observed ECM remodeling, may serve as a key pathological mechanism underlying sevoflurane‐induced neurotoxicity and cognitive deficits.

### 2.3 Cellular Localization Profiling of WFA‐Labeled PNN in the Hippocampal CA2 Region

First, we confirmed the robust expression of WFA‐labeled PNNs in the hippocampal CA2 subfield. To identify the specific cell types ensheathed by these specialized ECM structures, we characterized the cellular localization profile of PNNs in the CA2 region. As shown in Figure 3A, double immunofluorescence staining revealed that WFA immunoreactivity was highly colocalized with the pan‐neuronal marker NeuN, whereas only minimal colocalization was detected with canonical glial lineage markers, including glial fibrillary acidic protein (GFAP; astrocytes), CC1 (oligodendrocytes), and ionized calcium‐binding adapter molecule 1 (Iba1; microglia). Quantitative analysis of colocalization events confirmed that 95.06% of the WFA‐positive structures were associated with NeuN‐positive neurons, while only 2.30% were linked to CC1‐positive oligodendrocytes, 1.00% to GFAP‐positive astrocytes, and 1.64% to other cell types (Figure 3B). These data demonstrate that PNNs in the hippocampal CA2 region are predominantly associated with neurons, and negligibly associated with glial cells.

**FIGURE 3:**
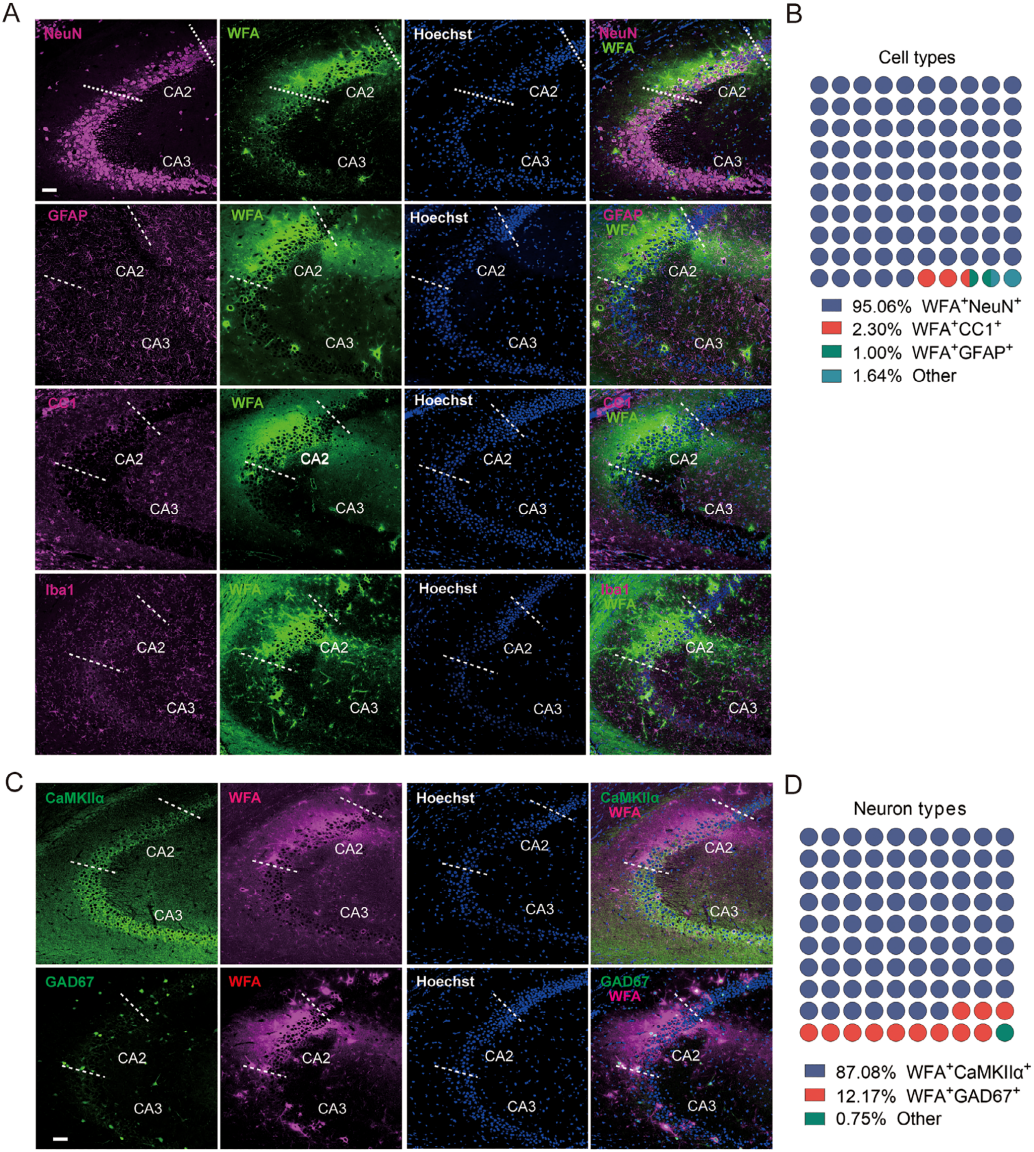
Characterization of the expression profile of WFA‐positive PNNs in the hippocampal CA2 region. (A) Representative co‐immunofluorescence of WFA‐positive PNNs with cell‐type‐specific markers in the CA2: NeuN (neurons), GFAP (astrocytes), CC1 (oligodendrocyte) and Iba1 (microglia). Scale bar = 50 µm. (B) Quantitative of the proportion of WFA‐positive PNNs associated with each cell type shown in (A). (C) Representative co‐immunofluorescence images of WFA‐positive PNNs with CaMK IIα (excitatory neuron) and GAD67‐GFP (inhibitory neuron) in the CA2. GAD67‐positive inhibitory neurons were identified by GFP fluorescence in GAD67‐GFP reporter mice. Scale bar = 50 µm. (D) Quantitative of the proportion of WFA‐positive PNNs associated with excitatory versus inhibitory neurons shown in (C). *n* = 3 mice per group.

To further delineate the specific neuronal subtypes ensheathed by the CA2 PNNs, we performed double immunofluorescence staining with well‐validated subtype‐specific neuronal markers. As illustrated in Figure 3C, WFA‐labeled PNNs robustly ensheathed both calcium/calmodulin‐dependent protein kinase II (CaMK2)‐positive excitatory neurons and glutamate decarboxylase 67 (GAD67)‐positive inhibitory neurons within the CA2 subfield. Quantitative analysis revealed that 87.08% of the neuron‐associated WFA‐positive PNNs ensheathed CaMKII‐positive excitatory neurons, while 12.17% surrounded GAD67‐positive inhibitory neurons, and only 0.75% were associated with other neuronal subtypes (Figure 3D). We next asked whether sevoflurane exposure preferentially disrupted PNNs associated with one of these neuronal populations. To address this, we quantified WFA fluorescence intensity specifically around CaMKII‐positive neurons and GAD67‐positive neurons in control and sevoflurane‐exposed mice. Following sevoflurane exposure, WFA signal around CaMKII‐positive excitatory neurons was significantly reduced compared with controls (Figure S5A,B). In contrast, WFA staining around GAD67‐positive inhibitory neurons, identified using GAD67‐GFP reporter mice, showed no significant change (Figure S5C, D). Collectively, these findings establish that CA2 PNNs predominantly ensheath excitatory neurons, identifying the cellular population most vulnerable to neonatal Sev‐induced PNN disruption in the CA2.

### 2.4 Repeated Neonatal Sevoflurane Exposure Induces Long‐Term Synaptic Impairment In Vivo and In Vitro via Dysregulating BDNF/TrkB Signaling

In the central nervous system plays a critical role, the ECM in regulating and stabilizing neural plasticity and synaptic functions during development and adulthood [34]. As a specialized subset of the ECM, PNNs function to constrain excessive synaptic remodeling and structural plasticity, thus preserving the long‐term stability of neuronal circuits [35]. Building on these established physiological roles, we next examined the development of excitatory synapses in the hippocampus 8 weeks after neonatal Sev exposure. Western blot analysis revealed significantly reduced expression of synaptic structural markers, PSD protein 95 (PSD95) and Synaptophysin, in the hippocampi of Sev‐exposed mice relative to Con animals (Figure 4A,B). Consistent with these molecular findings, Golgi staining demonstrated a marked decrease in dendritic spine density in Sev‐treated mice compared to that in Con mice (Figure 4C,D). At the ultrastructural level, transmission electron microscopy (TEM) analysis confirmed that Sev exposure significantly reduced the length, number, and thickness of PSD structures (Figure 4E–H).

**FIGURE 4:**
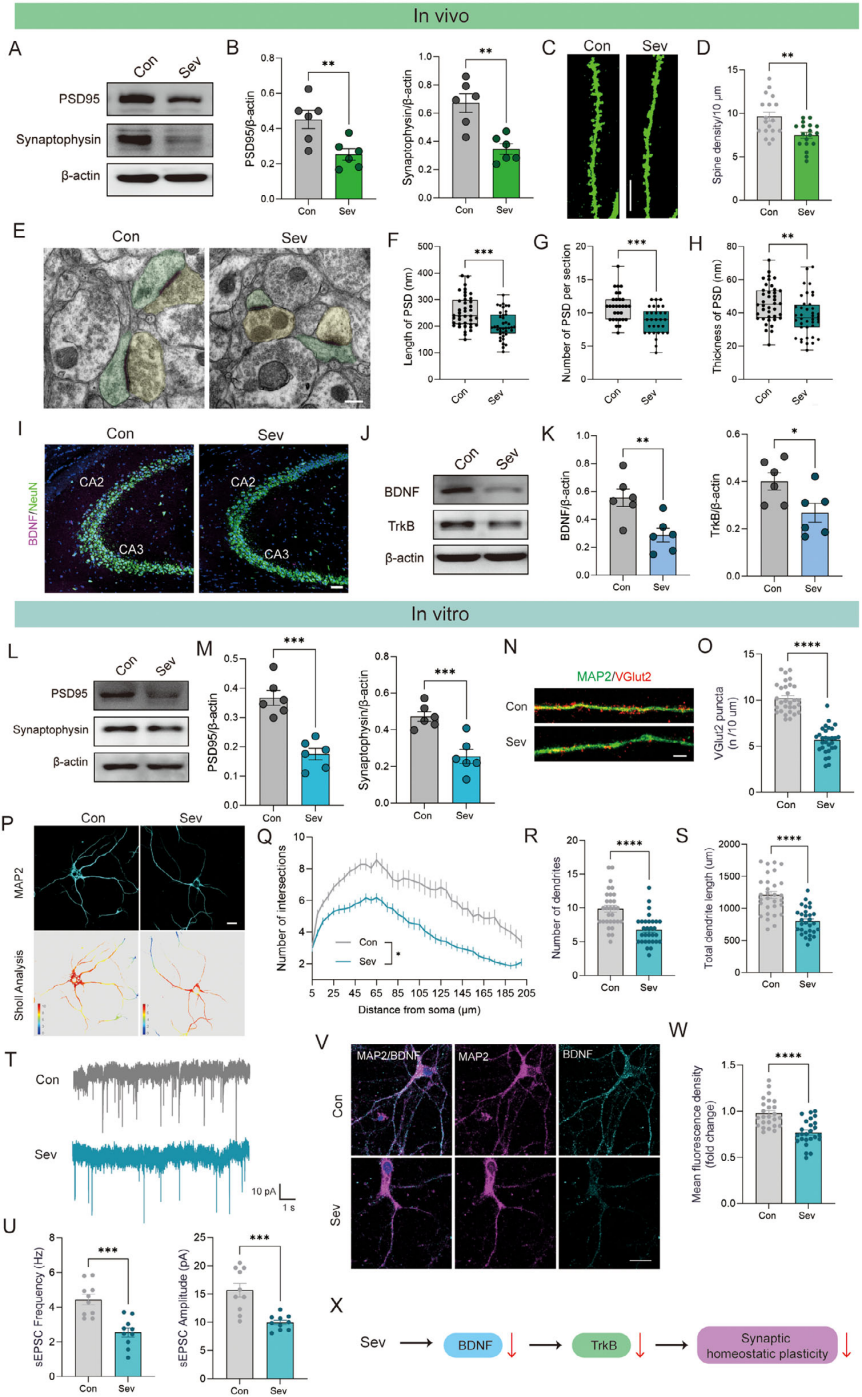
Impaired synapse development and reduced BDNF levels in Sev hippocampal neurons. (A, B) Western blotting and quantification of PSD95 and Synaptophysin protein levels in the hippocampus of Con and Sev mice. (C, D) Representative images of dendrites of hippocampal pyramidal neurons in Con and Sev groups. Scale bar = 10 µm. (E) Representative SEM micrographs of hippocampal synapses. Presynaptic terminals (yellow), postsynaptic terminals (green), and postsynaptic density (PSD, purple). Scale bar = 250 nm. (F–H) Quantification of PSD length (F), number of PSDs per section (G), and PSD thickness (H) in Con and Sev groups. (I) Representative immunofluorescence images of BDNF and NeuN in the CA2 and CA3 regions from Con and Sev mice. Scale bar = 50 µm. (J, K) Western blotting and quantification of BDNF and TrkB protein levels in the hippocampus of Con and Sev mice. (L, M) Western blotting and quantification of PSD95 and Synaptophysin protein levels in primary hippocampal neurons after Sev exposure. (N) Representative confocal microscopy images of MAP2 (green) and VGlut2 (red) colocalization in hippocampal neurons. Scale bar = 5 µm. (O) Quantification of VGlut2 puncta number per 10 µm in hippocampal neurons. (P) Representative MAP2‐labeled dendrites (top) and Sholl analysis (bottom) of Con and Sev neurons. Color gradients (red to blue) indicate dendritic intersections with increasing radial distance from the soma. Scale bar = 20 µm. (Q–S) Quantification of total dendritic length (Q), number of branch points (R), and Sholl intersections in Con and Sev neurons. (T, U) Representative traces (T) and quantification (U) of sEPSC frequency and amplitude in Con and Sev neurons. (V, W) Immunofluorescence staining images (V) and quantitative (W) of MAP2 and BDNF in Con and Sev neurons. Scale bar = 20 um. (X) A schematic model illustrating the BDNF‐dependent mechanism underlying Sev‐induced synaptic impairment. Data represent the mean ± SEM. *n* = 6 mice per group for in vivo experiments (A, B, J, K); *n* = 3 mice per group (E, F, G, H, I); *n* = 18 neurons from 3 mice per group (C, D). *n* = 6 independent cultures per group (L, M); *n* = 30–40 neurons per group (N, O, P, Q, R, S, V, W); *n* = 10 neurons per group (T, U). Statistical comparisons were conducted using unpaired Student's t‐test. ^*^ *p* < 0.05, ^**^ *p* < 0.01, ^***^ *p* < 0.001, ^****^ *P*< 0.0001.

BDNF signaling via its receptor, TrkB, is a critical regulator of synapse formation, synaptic plasticity. We examined hippocampal BDNF and TrkB expression and found that Sev‐exposed mice had significantly downregulated BDNF and TrkB protein levels consistent with synaptic dysfunction. This was confirmed by Western blotting and immunofluorescence assays. These findings suggest that impaired BDNF/TrkB signaling acts as an upstream mediator of Sev‐induced synaptic injury (Figure 4I–K).

To validate the in vivo observations of synaptic impairment and further explore its cellular mechanisms, we established primary hippocampal neuronal cultures. Sev treatment recapitulated the synaptic defects observed in vivo, with a significant reduction in the expression of PSD95 and Synaptophysin (Figure 4L,M). Consistent with these reductions in synaptic structural proteins, quantitative analysis of vesicular glutamate transporter 2 (VGlut2) puncta density (a marker for excitatory synapses—revealed a marked reduction in Sev‐treated neurons, confirming impaired synaptic assembly (Figure 4N,O). Functional and morphological assessments further demonstrated Sev‐induced neurotoxicity in primary hippocampal neurons. Sholl analysis showed simplified dendritic arborization with significantly reduced in dendritic branches and total dendritic length (Figure 4P–S). Whole‐cell patch‐clamp recordings revealed decreased frequency and amplitude of spontaneous excitatory postsynaptic currents (sEPSCs), directly indicating synaptic transmission dysfunction (Figure 4T,U). Notably, in vitro Sev treatment also significantly suppressed BDNF expression, consistent with in vivo downregulation of BDNF/TrkB signaling (Figure 4V,W).

Collectively, these data indicate that neonatal Sev exposure triggers long‐term synaptic impairment both in vivo and in vitro, likely via inhibition BDNF/TrkB signaling, thereby disrupting synaptic homeostatic plasticity (Figure 4X).

### 2.5 Hapln1 Rescues PNN Integrity and Reverses Cognitive Deficits Following Neonatal Sevoflurane Exposure

To clarify whether PNNs regulate Sev‐induced cognitive impairment, we stereotaxically injected chondroitinase ABC (ChABC) into the hippocampal CA2 region of adult mice on postnatal day 55 (P55) (Figure S6A). ChABC, a bacterial glycosidase, cleaves CS‐GAG chains from PNN core proteoglycans [33]. Confocal immunofluorescence showed abundant WFA‐positive PNNs and WFA‐PCP4 co‐localization in control mice treated with phosphate‐buffered saline (Con+PBS). In contrast, ChABC treatment nearly eliminated WFA staining, confirming effective PNN disruption (Figure S6B–D). These results established ChABC as a valid tool for investigating the functional consequences of PNN disruption in Sev‐induced neurotoxicity. Next, we performed an in vivo rescue experiment using targeted overexpression of Hapln1 to restore PNN integrity. After neonatal Sev exposure on P6–P8, we stereotaxically injected Cre‐dependent rAAV‑DIO‑Hapln1 (for Hapln1 overexpression) or control rAAV‑DIO‑EGFP into the hippocampal CA2/3 region of Camk2‑Cre mice (Figure 5A,B) (Figure S7). Consistent with our previous results, Sev exposure reduced WFA‐labeled PNN intensity by approximately 50% in the CA2 region of Dio‑EGFP control mice (Figure 5C,D). Notably, WFA‐labeled PNN intensity was significantly higher in Dio‑Hapln1 + Sev mice than in the Dio‑EGFP + Sev group, confirming that Hapln1 overexpression rescues PNN integrity from Sev‐induced damage.

**FIGURE 5:**
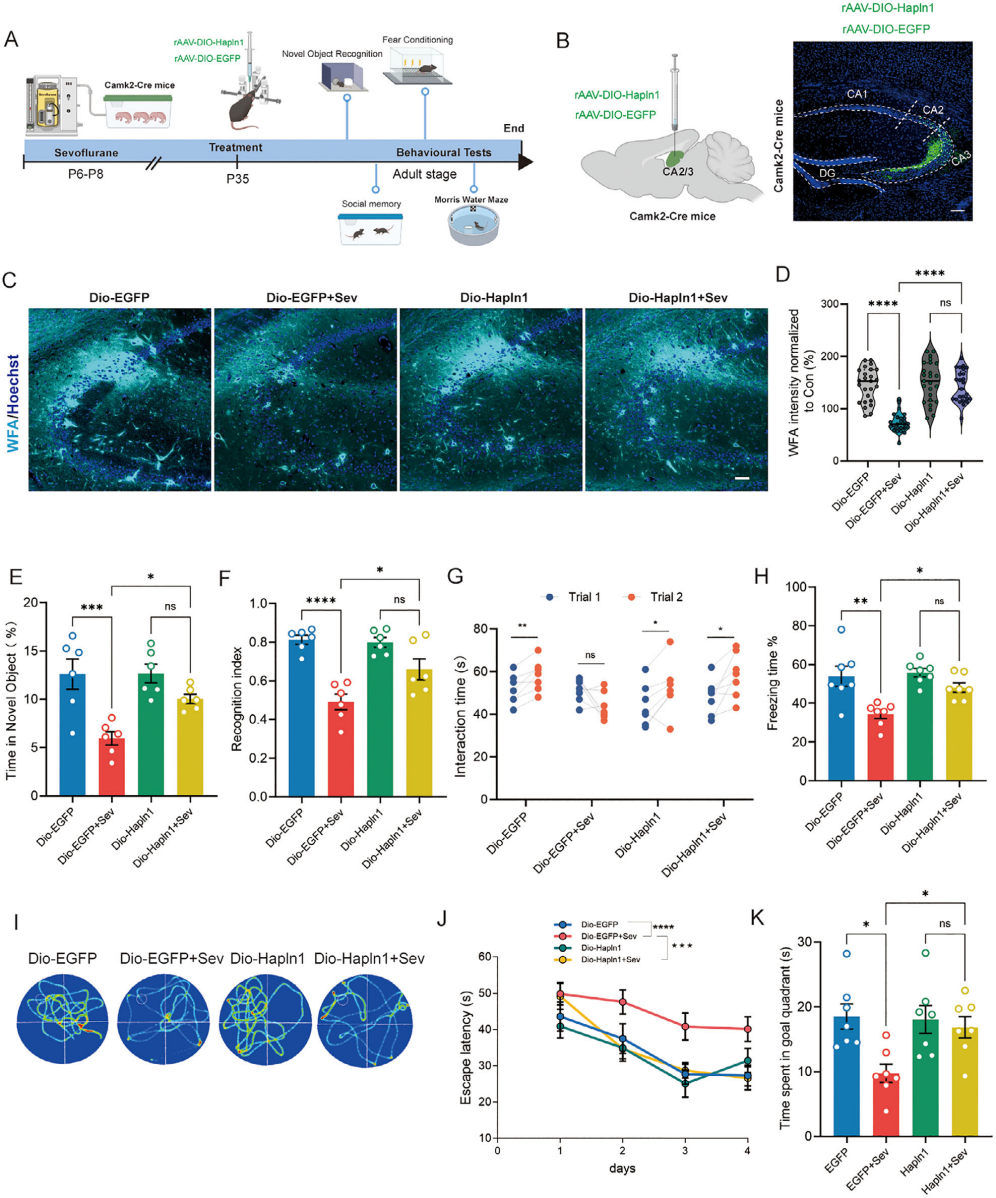
Hapln1 regulates PNNs remodeling and rescues sevoflurane‐induced cognitive impairment. (A) Schematic of the experimental design. (B) Representative image showing viral expression (EGFP) after stereotaxic injection of rAAV‐Dio‐Hapln1 or rAAV‐Dio‐EGFP into the CA2/3 region of Camk2‐Cre mice. Scale bar = 100 µm. (C, D) Representative WFA staining (C) and quantitative (D) of PNNs in the CA2 region across four groups: Dio‐EGFP, Dio‐EGFP+Sev, Dio‐Hapln1, and Dio‐Hapln1+Sev. Scale bar = 50 µm. (E, F) Novel object exploration time (E) and recognition index (F) across the four groups. (G) Social memory interaction times across the four groups. Each data point represents an individual mouse. (H) Freezing time percentage in the fear conditioning tests across the four groups. (I–K) Morris water maze test across the four groups: escape latency during training (J), time in target quadrant (K). Dio‐EGFP: Con mice receiving rAAV‐DIO‐EGFP. Dio‐EGFP+Sev: Mice receiving rAAV‐DIO‐EGFP plus Sev exposure. Dio‐Hapln1: Con mice receiving rAAV‐DIO‐Hapln1. Dio‐Hapln1+Sev: Mice receiving rAAV‐DIO‐Hapln1 plus Sev exposure. Data represent the mean ± SEM. *n* = 6–7 mice per group. Statistical comparisons were performed using one‐way ANOVA followed by Tukey's post hoc test (D, E, F, G, H, K) or two‐way repeated‐measures ANOVA followed by Sidak's post hoc test (J). ^*^ *P*< 0.05, ^**^ *p* < 0.01, ^***^ *p* < 0.001, ^****^ *p* < 0.0001.

Next, we evaluated the impact of ChABC‐mediated PNN degradation on Sev‐induced cognitive impairment (Figure S8A). In the novel object recognition test, Sev+PBS mice showed reduced novel object exploration and recognition index. ChABC alone caused similar deficits and failed to reverse Sev‐induced impairment, with ChABC+Sev mice exhibiting deficits comparable to those of Sev+PBS mice (Figures S8B,C). Regarding social memory, Con+PBS mice had increased Trial 2 interaction time, while Sev+PBS, ChABC, and ChABC+Sev mice showed no trial‐dependent difference (Figure S8D). In terms of fear conditioning, both Sev exposure and ChABC treatment alone reduced freezing time (Figure S8E). Collectively, these findings confirm that PNN degradation recapitulates Sev‐induced cognitive dysfunction, demonstrating the protective effects of intact PNNs against Sev neurotoxicity.

To further validate PNN‐mediated protection, we assessed whether Hapln1‐mediated PNN restoration reversed Sev‐induced cognitive impairment. In the novel object recognition test, Sev‐exposed DIO‐EGFP mice showed significantly reduced novel object exploration time and recognition index, reflecting recognition memory deficits that were effectively reversed by Hapln1 overexpression (Figure 5E,F). In the social memory test, Dio‐EGFP control mice had significantly increased interaction time with a novel conspecific during Trial 2 compared to the familiar conspecific in Trial 1, which is a hallmark of intact social recognition memory. In contrast, Sev‐exposed Dio‐EGFP mice failed to exhibit this preference, indicating severe social memory impairment. Hapln1 overexpression effectively restored social memory in Sev‐exposed mice, as reflected by the reinstated preference for the novel conspecific in Trial 2 (Figure 5G). Consistent with these findings, Sev‐exposed Dio‐EGFP mice showed significantly reduced freezing time in the fear conditioning test, reflecting impaired contextual fear memory. Hapln1 overexpression effectively restored the freezing behavior in Sev‐exposed mice (Figure 5H). Finally, in the Morris water maze test, Sev‐exposed Dio‐EGFP mice showed prolonged escape latencies during training and reduced time in the target quadrant during the probe trial, reflecting impaired spatial learning and memory. Hapln1 overexpression significantly improved these outcomes, enhancing both learning efficiency and spatial memory retention in Sev‐exposed mice (Figure 5I–K).

Collectively, these in vivo rescue findings indicate that Hapln1 downregulation contributes to Sev‐induced PNN disruption and consequent cognitive dysfunction, supporting Hapln1 as a potential therapeutic target for alleviating anesthetic‐induced developmental neurotoxicity.

### 2.6 Hapln1 Overexpression Reverses Sev‐Induced Synaptic Impairment and Restores Hippocampal BDNF/TrkB Signaling In Vivo

To investigate how PNN integrity is related to Sev‐induced synaptic dysfunction, we validated two complementary in vitro models using primary hippocampal neurons (Figure S9). Cultures were derived from embryonic day 15 (E15) mouse embryos (Figure S9A); consistent with our in vivo observations, Sev significantly reduced the number of WFA‐positive neurons (Figure S9B,C), confirming that Sev directly targets PNNs rather than causing overt neuronal toxicity. To isolate the specific effect of PNN loss, we used ChABC, which cleaves the PNN core proteoglycan side chains. Time‐course experiments (2–48 h) showed progressive, time‐dependent loss of WFA signal, with near‐complete PNN clearance by 48 h (Figure S9D), and ChABC‐treated cultures had significantly fewer WFA‐positive neurons than PBS controls at this endpoint (Figure S9E,F), validating this as a reliable PNN depletion model.

Next, we evaluated whether Hapln1‐mediated PNN restoration rescued Sev‐induced synaptic damage in vivo. Western blot analysis revealed that neonatal Sev exposure significantly downregulated the expression of the postsynaptic marker PSD95 and presynaptic marker Synaptophysin in the hippocampus of DIO‐EGFP control mice (Figure 6A–C); notably, these reductions were effectively reversed by targeted Hapln1 overexpression (Figure 6A–C). Consistently, Golgi staining demonstrated that Sev exposure caused a marked decrease in dendritic spine density in hippocampal neurons, which was significantly rescued by Hapln1 overexpression (Figure 6D,E). TEM analysis at the ultrastructural level further confirmed that Sev exposure significantly reduced the number, length, and thickness of PSD structures, and Hapln1 overexpression effectively restored these PSD parameters to control levels (Figure 6F–I). Additionally, Sev exposure significantly downregulated the expression of BDNF and its receptor TrkB, key regulators of synaptic plasticity, and this downregulation was also rescued by Hapln1 overexpression (Figure 6J–L).

**FIGURE 6:**
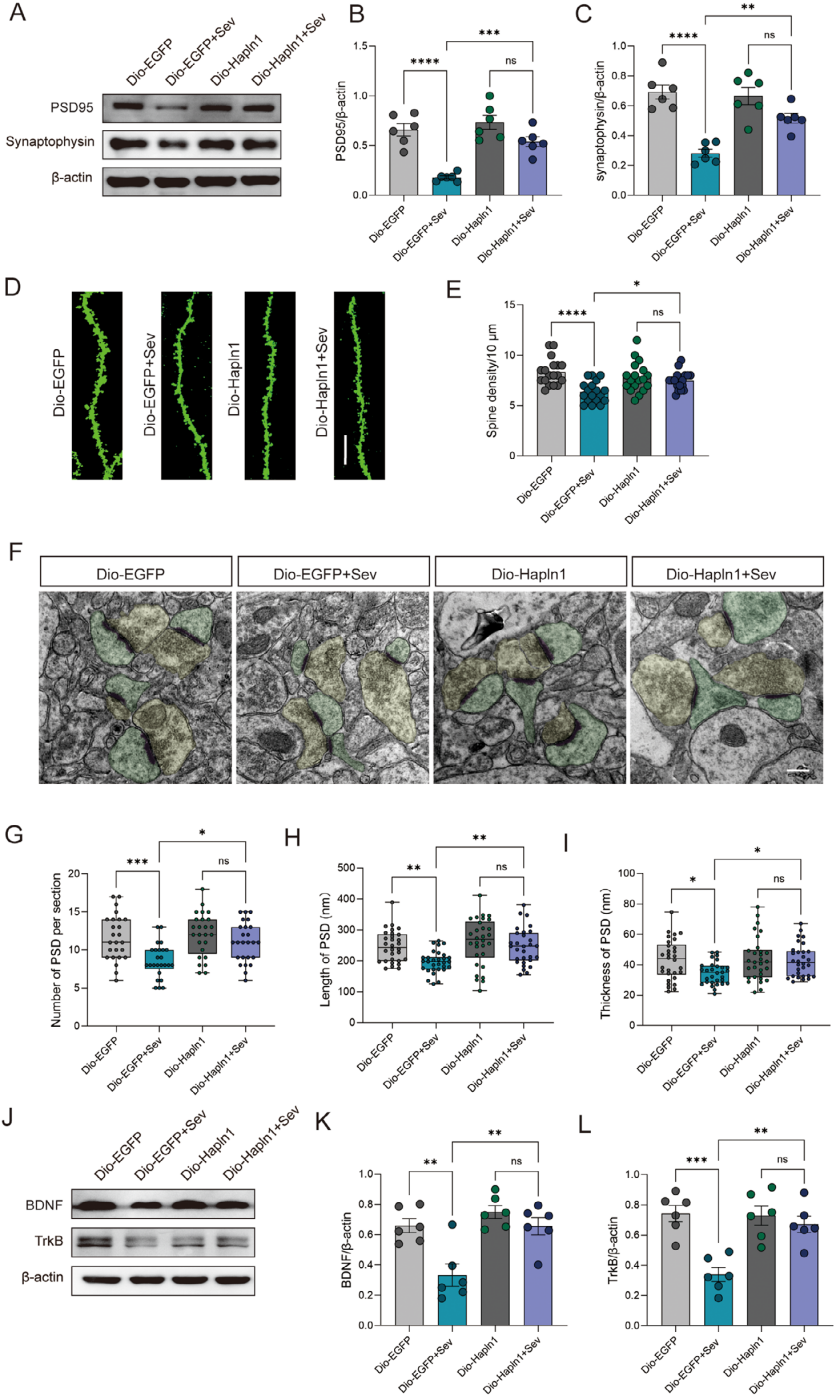
Hapln1 overexpression restores hippocampal synaptic ultrastructure and BDNF‐TrkB signaling disrupted by sevoflurane exposure. (A–C) Representative Western blots (A) and quantification of PSD95 (B) and Synaptophysin (C) protein levels in the hippocampus across four groups: Dio‐EGFP, Dio‐EGFP+Sev, Dio‐Hapln1, and Dio‐Hapln1+Sev groups. (D, E) Golgi staining (D)and quantification of dendrite spine density (E) across the four groups. Scale bar = 10 µm. (F–I) Synaptic ultrastructure in the hippocampus across the four groups. (F) Representative TEM images of synapses. Scale bar = 250 nm. Quantification of the number of PSDs (G), PSD length (H), and PSD thickness (I). (J–L) Representative Western blots (J) and quantification of BDNF (K) and TrkB (L) protein levels in the hippocampus across the four groups. Data represent the mean ± SEM. *n* = 6 mice per group (A–C, J–L); *n* = 18 neurons from 3 mice per group (D, E); *n* = 3 mice per group (F, G, H, I). Statistical comparisons were performed using One‐way ANOVA followed by Tukey's post hoc test. ^*^ *P*< 0.05, ^**^ *p* < 0.01, ^***^ *p* < 0.001, ^****^ *p* < 0.0001.

Next, we investigated whether PNN degradation recapitulates Sev‐induced synaptic impairment using ChABC (Figure S10A–H). ChABC treatment significantly reduced Hapln1 expression in vivo, confirming efficient PNN degradation (Figure S10A,B). Western blot analysis showed that ChABC treatment markedly downregulated PSD95, Synaptophysin, BDNF, and TrkB expression in the hippocampus, mirroring the effects of Sev exposure (Figure S10C,D,I–K). TEM analysis further revealed that ChABC treatment significantly decreased the number, length, and thickness of PSD structures, consistent with Sev‐induced synaptic damage (Figure S10E,F). To confirm the direct effects of PNN degradation on neuronal development and synaptic function, we performed in vitro experiments using primary hippocampal neurons. Sholl analysis demonstrated that ChABC treatment significantly reduced dendritic complexity, total dendritic length, and the number of dendrites in cultured neurons (Figure S10L–O). Whole‐cell patch‐clamp recordings further showed that ChABC treatment significantly decreased both the frequency and amplitude of sEPSCs, indicating impaired synaptic function following PNN degradation (Figure S10P–R).

Collectively, these results demonstrate that intact PNNs are critical for maintaining hippocampal synaptic structure and function. Hapln1‐mediated PNN restoration rescued Sev‐induced synaptic damage, whereas PNN degradation by ChABC recapitulated Sev‐induced synaptic impairment, establishing PNN integrity as a key protective factor against Sev‐induced developmental neurotoxicity via the modulation of synaptic structure and the BDNF/TrkB signaling pathway.

### 2.7 Neonatal Sev Exposure Upregulates Hippocampal Mmp9 Expression by Attenuating H3K27me3‐Mediated Transcriptional Repression

Next, we examined the expression of MMP9, a key protease that mediates PNN breakdown, in the hippocampus following neonatal Sev exposure. Western blot analysis revealed a significant upregulation of both active MMP9 and pro‐MMP9 in Sev‐exposed mice relative to controls (Figure 7A,B). Enzyme‐linked immunosorbent assay (ELISA) further confirmed a marked increase in total hippocampal MMP9 levels following Sev treatment (Figure 7C). Immunofluorescence staining showed localization of MMP9 to NeuN‐positive hippocampal neurons, with distinctly higher fluorescence intensity detected in Sev‐exposed animals (Figure 7D).

**FIGURE 7:**
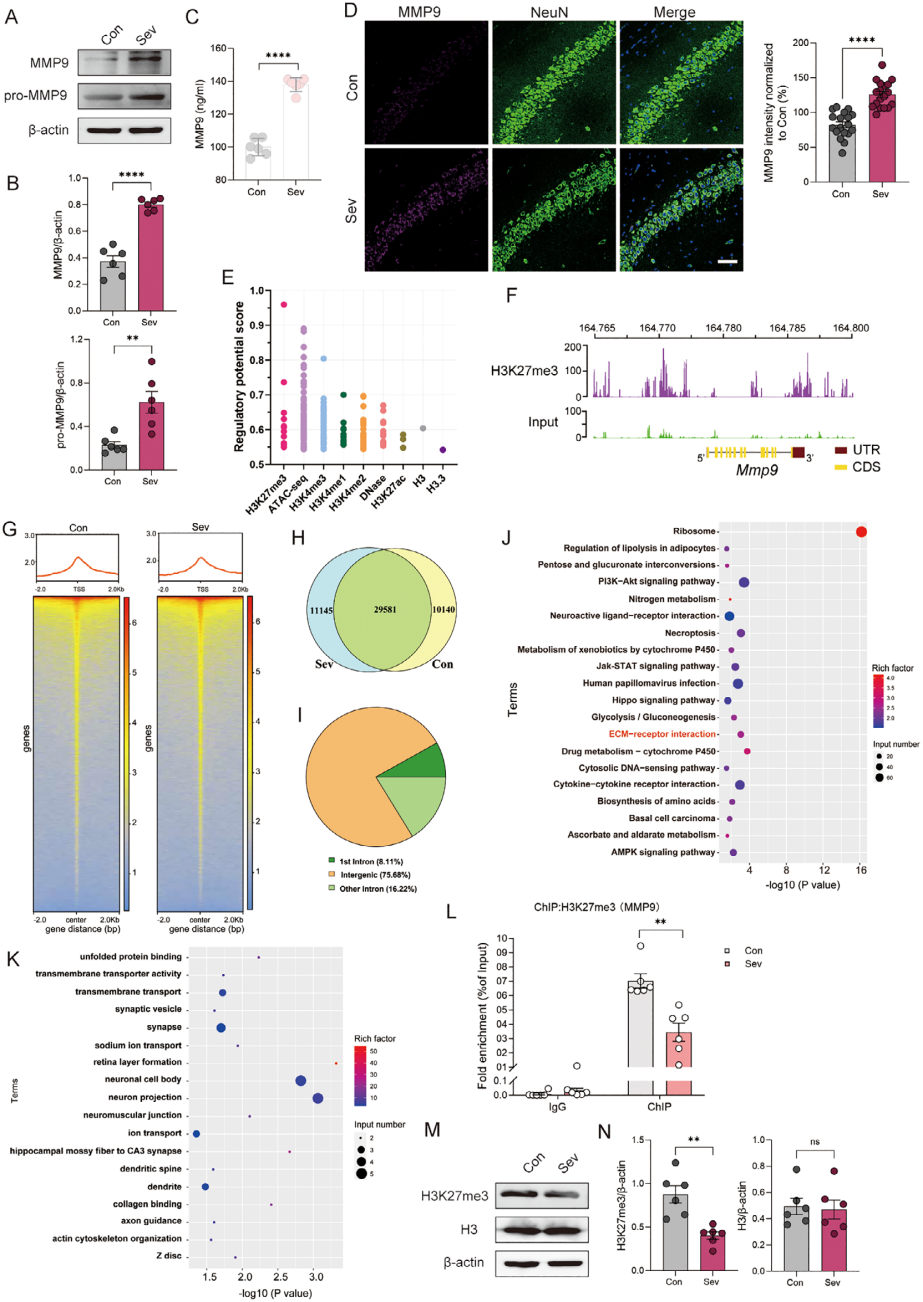
Sevoflurane exposure upregulates hippocampal MMP9 via epigenetic H3K27me3 modification in mice. (A, B) Representative Western blots (A) and quantification (B) of pro‐MMP9 and mature MMP9 protein levels in the hippocampus of Con and Sev ‐exposed mice. (C) ELISA quantification of MMP9 concentration in the hippocampus of Con and Sev mice. (D) Immunofluorescence staining and quantitative of MMP9 in the hippocampus of Con and Sev mice. Scale bar = 50 µm. (E) Histone modification regulatory scores of *Mmp9*‐targeting transcription factors, analyzed using the Cistrome DB Toolkit. (F) Genomic annotattion and motif analysis of H3K27me3 modifications identified by ChIP‐seq (dataset GSM1570789). (G) Heatmap showing differential H3K27me3 enrichment around transcription start sites (TSS). (H) Venn diagram showing H3K27me3 peaks between Con and Sev groups. (I) Pie chart showing genomic distribution of differentially H3K27me3‐modified regions (first intron, intergenic, other intron). (J, K) KEGG pathway (J) and GO enrichment (K) analyses of genes with differential H3K27me3 modification. (L) ChIP‐qPCR validation of H3K27me3 enrichment at the Mmp9 locus in Con and Sev mice, with IgG as a negative control. (M, N) Representative Western blots (M) and quantification (N) of global H3K27me3 and total H3 in the hippocampus of Con and Sev mice. Data represent the mean ± SEM. *n* = 6 mice per group (A–C, L–N); *n* = 3 mice per group (D, G, H–K). Statistical comparisons between two groups were conducted using an unpaired Student's t‐test. ^**^ *p* < 0.01, ^****^ *p* < 0.0001.

Multiple histone modifications have been implicated in regulating *Mmp9* mRNA expression [36]. Among these, H3K27me3 exhibited the highest transcriptional regulatory score for *Mmp9* (Figure 7E) based on analysis using the Cistrome DB Toolkit (http://dbtoolkit.cistrome.org). H3K27me3 is widely implicated in modulating neuronal activity and mediating the transcriptional regulation of Mmp9. Reanalysis of public H3K27me3 chromatin immunoprecipitation sequencing (ChIP‐seq) data (GSM1570789) further showed that H3K27me3‐enriched peaks were predominantly located in the Mmp9 promoter (Figure 7F).

To verify this, we performed H3K27me3 ChIP‐seq on hippocampal tissue 3 days after Sev exposure. ChIP‐seq heatmaps and Venn diagrams further revealed distinct patterns of chromatin accessibility between Con and Sev‐exposed mice, with most peaks distributed in the intergenic and intronic regions (Figure 7G–I). KEGG analysis of the differential ChIP‐seq peaks showed marked enrichment in the ECM‐receptor interaction pathway (Figure 7J). Gene Ontology (GO) enrichment further highlighted synaptic‐related terms including synapse, dendritic spine, and hippocampal mossy fiber to CA3 synapse, supporting a close link between epigenetic alterations and impaired synaptic integrity (Figure 7K).

Finally, ChIP‐qPCR was performed to validate our ChIP‐seq results, which confirmed significantly reduced H3K27me3 enrichment at the *Mmp9* promoter in Sev‐exposed mice (Figure 7L). Western blotting further showed that Sev exposure lowered global H3K27me3 levels without altering total histone H3 expression (Figure 7M). Additional ChIP‐qPCR verified the reduction in H3K27me3 at the Mmp9 locus, with no significant change in H3 enrichment (Figure 7N).

We next asked whether the loss of H3K27me3 was required for Sev‐induced Mmp9 upregulation and PNN disruption. Primary hippocampal neuronal cultures were pretreated with GSK‐J4, a selective H3K27me3 demethylase inhibitor, before Sev exposure. GSK‐J4 (0.5 µM) pretreatment significantly attenuated Sev‐driven Mmp9 upregulation. In parallel, WFA staining showed that PNN loss was largely prevented by GSK‐J4 (Figure S11A–D). Together, these results support a causal role for H3K27me3 reduction in Mmp9 upregulation and subsequent PNN degradation following Sev exposure.

### 2.8 Pharmacological Inhibition of MMP9 by SB‐3CT Rescues Sev‐Induced PNN Degradation and Cognitive Impairment

We next investigated whether SB‐3CT mediated MMP9 inhibition could mitigate Sev‐induced cognitive deficits and PNN disruption in mice by intraperitoneally administering the selective MMP9 inhibitor SB‐3CT (25 mg/kg) within 30 min of neonatal Sev exposure [37]. Neonatal Sev exposure caused persistent cognitive impairments in adulthood. In the novel object recognition test, Sev‐exposed mice spent significantly less time exploring the novel object and had a lower recognition index than Con mice, and both deficits were effectively rescued by SB‐3CT treatment (Figure 8B,C). Sev exposure also impaired fear conditioning memory, as shown by reduced freezing time, and disrupted social memory. Sev‐exposed mice showed no significant difference in interaction time between the two trials, confirming social memory impairment, which was fully reversed by SB‐3CT treatment (Figure 8D,E). In the Morris water maze, Sev‐exposed mice exhibited prolonged escape latencies across training days and spent significantly less time in the target quadrant during the probe trial, reflecting spatial learning and memory deficits that were markedly ameliorated by SB‐3CT administration (Figure 8F,G).

**FIGURE 8:**
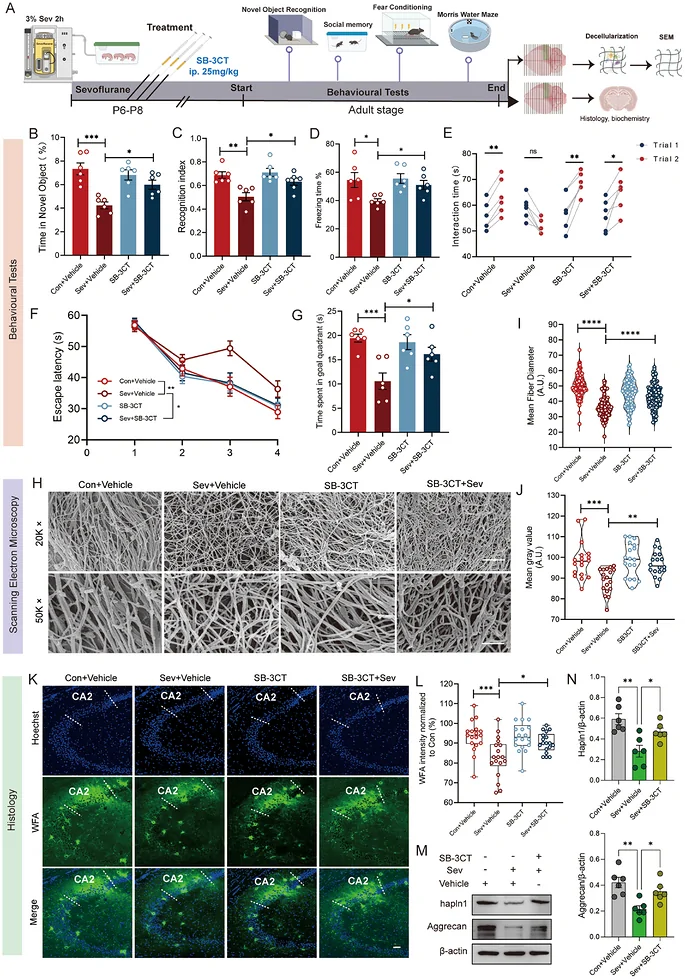
Pharmacological inhibition of MMP9 by SB‐3CT rescues sevoflurane‐induced cognitive impairment and PNNs deficits. (A) Schematic of the experimental design. (B, C) Novel object recognition: exploration time (B) and recognition index (C) across four groups: Con + Vehicle, Sev + Vehicle, SB‐3CT, and Sev + SB‐3CT groups (*n* = 6 mice per group). (D) Freezing time percentage in the fear conditioning test across the four groups. (E) Social memory interaction times across the four groups. Each data point represents an individual mouse. (F, G) Morris water maze test: escape latency (F) and time spent in goal quadrant (G). (H–J) SEM imaging and quantification of hippocampal ECM ultrastructure across the four groups. Scale bars = 1.2 µm (20K×), 500 nm (50K×). (K, L) WFA staining (K) and quantification (L) of hippocampal PNNs across the four groups. Scale bar = 50 µm. (M, N) Western blotting and quantification of Hapln1 and aggrecan protein levels across the three groups. Data represent the mean ± SEM. n = 6 mice per group (B–G, K–N); n = 3 mice per group (H–J). Statistical comparisons were performed using One‐way ANOVA followed by Tukey's post hoc test (B–E, G, I, J, L–N), and two‐way repeated‐measures ANOVA followed by Sidak's post hoc test (F). ^*^ *P*< 0.05, ^**^ *p* < 0.01, ^***^ *p* < 0.001, ^****^ *p* < 0.0001.

Next, we assessed the structural integrity of PNNs, which are key regulators of cognitive function. SEM analysis of decellularized hippocampal tissue showed that neonatal Sev exposure induced a marked decrease in both the mean diameter and mean gray value of the ECM fiber network, consistent with the effects of PNN degradation. Treatment with SB‐3CT effectively restored these structural metrics, confirming the rescue of PNN architecture (Figure 8H–J). Consistently, immunofluorescence staining with WFA revealed a significant decrease in WFA fluorescence intensity in the hippocampal CA2 region of Sev‐exposed mice, which was markedly elevated by SB‐3CT co‐treatment (Figure 8K,L). Western blotting further verified that Sev exposure significantly downregulated the expression of key PNN core components, Hapln1 and Aggrecan, and SB‐3CT treatment effectively reversed these reductions (Figure 8M,N). This confirms that MMP9 inhibition preserves PNN structural integrity and thereby rescues cognitive function from Sev‐induced neurotoxicity.

### 2.9 Pharmacological Inhibition of MMP9 by SB‐3CT Rescues Sev‐Induced Synaptic Dysfunction In Vivo and In Vitro

Immunoblotting revealed that Sev exposure significantly reduced hippocampal levels of the synaptic proteins PSD95 and Synaptophysin, while co‐treatment with SB‐3CT effectively reversed these reductions (Figure 9A–C). Consistent with this finding, Sev‐exposed mice exhibited decreased dendritic spine density in the hippocampus, which was restored by SB‐3CT administration (Figure 9D,E). Ultrastructural analysis using TEM further confirmed that SB‐3CT treatment ameliorated Sev‐induced impairments in PSD structure, including increases in PSD number, length, and thickness (Figure 9F–H). Additionally, SB‐3CT significantly reversed Sev‐induced downregulation of BDNF and TrkB protein levels in the hippocampus (Figure 9I,J).

**FIGURE 9:**
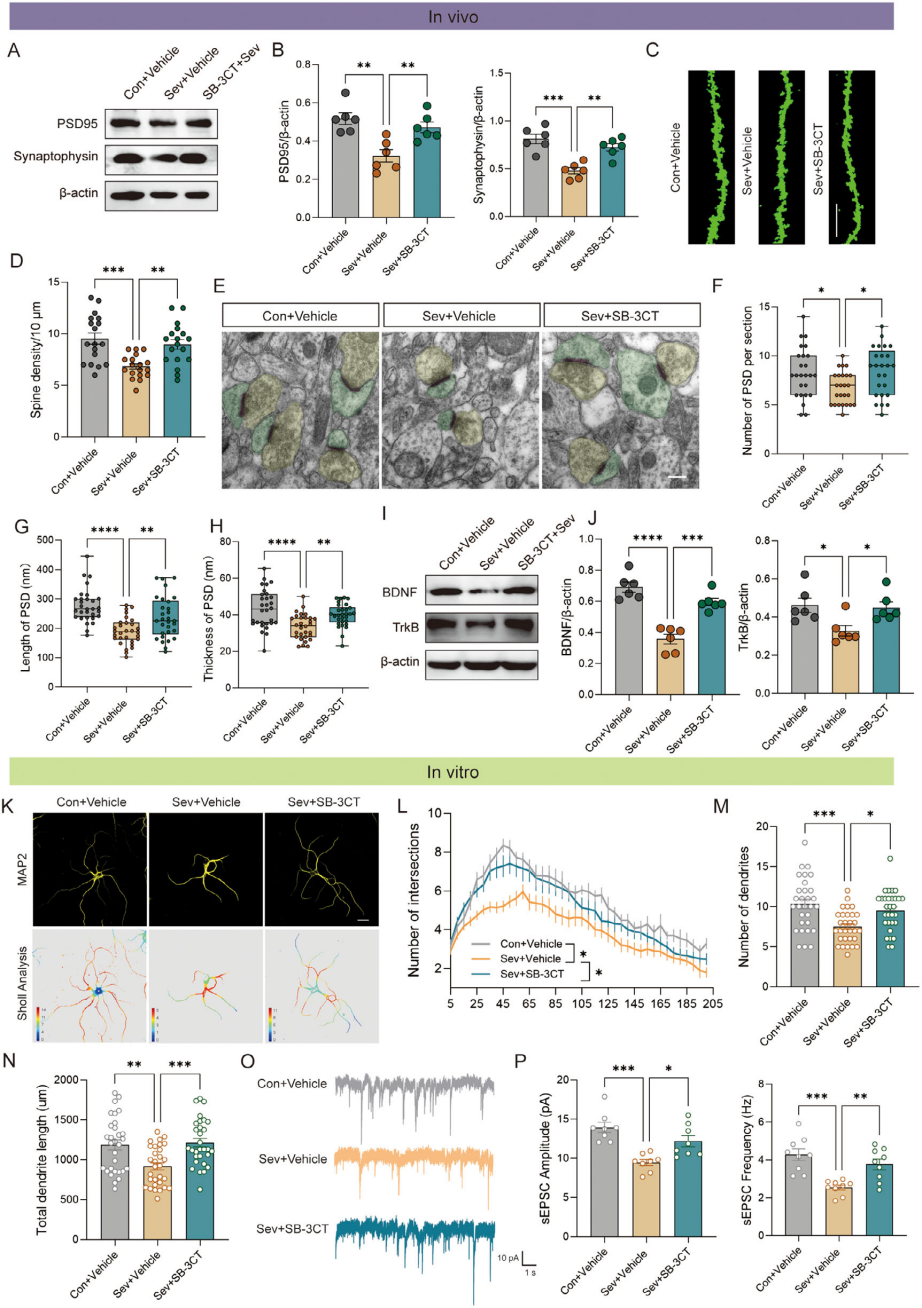
SB‐3CT rescues sevoflurane‐induced synaptic impairment and restores BDNF–TrkB signaling in the hippocampal. (A, B) Western blotting and quantification of PSD95 and synaptophysin protein levels in the hippocampus across three groups: Con + Vehicle, Sev + Vehicle, and Sev + SB‐3CT. (C, D) Golgi staining (C) and quantification of dendritic spine density (D) in CA2 pyramidal neurons across the three groups. Scale bar = 10 µm. (E) Representative SEM micrographs of hippocampal synapses. Presynaptic terminals (yellow), postsynaptic terminals (green), and postsynaptic density (PSD, purple) are pseudocolored. Scale bar = 250 nm. (F–H) Quantification of PSD number per section (F), PSD length (G), and PSD thickness (H) across the three groups. (I, J) Western blotting (I) and quantification (J) of BDNF and TrkB protein levels in the hippocampus across the three groups. (K–N) Representative MAP2 immunofluorescence staining (top) and Sholl analysis (bottom) of primary hippocampal neurons across the three groups (K). Color gradients (red to blue) indicate dendritic intersections with increasing radial distance from the soma. Scale bar = 20 µm. Quantification of Sholl intersections (L), number of dendrites (M), total dendritic length (N). (O, P) Representative traces (O) and quantification (P) of sEPSCs frequency and amplitude in primary hippocampal neurons across the three groups. Data represent the mean ± SEM. n = 6 mice per group (A, B, I, J); n = 3 mice per group (E, F, G, H); n = 18 neurons from 3 mice per group (C, D); n = 30–40 neurons per group (K, L, M, N); n = 8–9 neurons per group (O, PP). Statistical comparisons were performed using One‐way ANOVA followed by Tukey's post hoc test (B, D, F–H, J, L–P). ^*^ *P*< 0.05, ^**^ *p* < 0.01, ^***^ *p* < 0.001, ^****^ *p* < 0.0001.

Consistent with the in vivo findings, Sev exposure induced morphological retraction of microtubule‐associated protein 2 (MAP2)‐positive neurons and reduced dendritic complexity in primary hippocampal neurons, as evidenced by Sholl analysis. Treatment with SB‐3CT significantly reversed these morphological deficits, increasing the number of intersections and total dendritic length (Figure 9K–N). Electrophysiological recordings demonstrated that Sev exposure decreased both the amplitude and frequency of miniature excitatory postsynaptic currents (mEPSCs), whereas SB‐3CT treatment significantly restored these synaptic transmission parameters (Figure 9O–P).

To further test whether restoring BDNF signaling is sufficient to rescue Sev‐induced synaptic deficits, we applied exogenous BDNF (10 ng/mL) to primary hippocampal neurons within 30 min after Sev exposure. BDNF treatment significantly increased the protein levels of PSD95 and synaptophysin, which were reduced by Sev exposure (Figure S11E,F). These data indicate that BDNF depletion contributes to the synaptic dysfunction caused by Sev, and that replenishing BDNF can ameliorate these deficits.

## 3 Discussion

The results of this study delineate a definitive mechanistic cascade (graphically summarized in Figure 10). Neonatal Sev exposure induces a targeted reduction in the repressive histone modification H3K27me3 at the *Mmp9* promoter in hippocampal CA2 excitatory pyramidal neurons. This epigenetic derepression markedly elevates *MMP9* transcription and proteolytic activity, which in turn triggers the robust degradation of PNNs. As specialized Hapln1–aggrecan‐enriched ECM compartments, PNNs act as critical upstream regulators of BDNF/TrkB signaling homeostasis. The loss of PNN structural integrity disrupts the local neural microenvironment, limits extracellular BDNF bioavailability, impairs synaptic TrkB distribution, and ultimately suppresses neurotrophic signal transduction. Functionally, such a disruption drives maladaptive synaptic plasticity, weakens excitatory synaptic transmission, and reduces dendritic spine density. This sequential structural and functional damage collectively leads to persistent cognitive and social behavioral impairments. In this context, our findings establish PNN dysfunction as a vital extracellular mediator that translates transient sevoflurane stimulation into long‐term synaptic and circuit defects in the developing brain.

**FIGURE 10:**
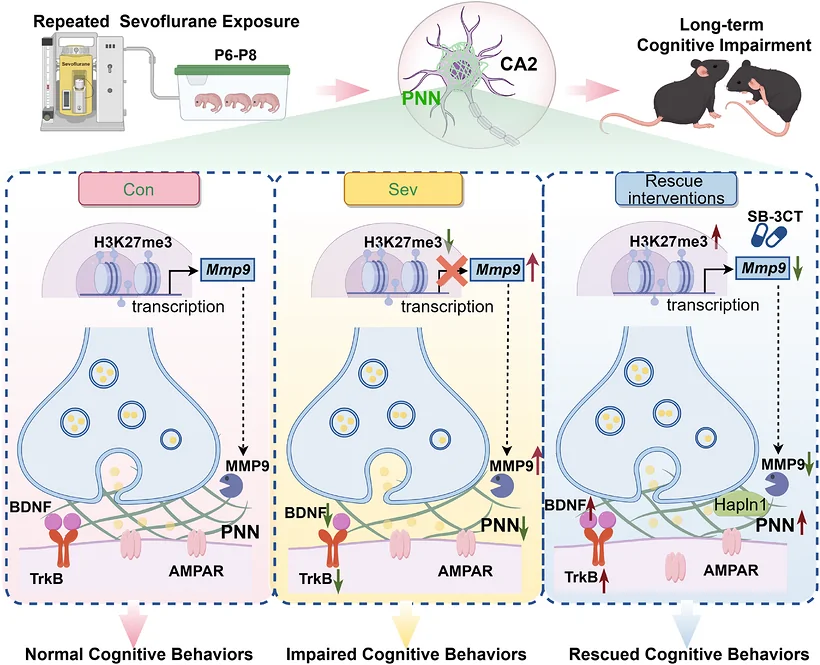
Working model of Sev‐induced long‐term cognitive impairment and rescue in the hippocampal CA2 region. Repeated neonatal sevoflurane exposure reduces the repressive histone modification H3K27me3, de‐repressing Mmp9 transcription. Elevated MMP9 drives perineuronal net (PNN) degradation in CA2 excitatory neurons, disrupting BDNF/TrkB signaling and AMPAR function, leading to persistent cognitive impairment. Targeted interventions (MMP9 inhibition via SB‐3CT or Hapln1 overexpression) restore PNN integrity, rescue synaptic function, and reverse sevoflurane‐induced cognitive deficits, identifying the H3K27me3‐MMP9‐PNN axis as a key mediator of developmental neurotoxicity and a promising therapeutic target. Illustrated using Figdraw (www.figdraw.com) and Adobe Illustrator. Copyright code: SUWUY4c411.

Preclinical evidence has consistently associated early‐life Sev exposure with elevated risk of neurodevelopmental disorders and long‐term cognitive dysfunction [9, 38]; however, epidemiological findings in human infants remain heterogeneous and have not established a definitive causal link, leaving pediatric anesthetic safety an ongoing area of clinical concern [4]. Mechanistically, previous studies have predominantly focused on intracellular pathological events, including oxidative stress, neuronal apoptosis, and neuroplastic dysfunction [7, 39]. Accumulating evidence from studies on inhaled anesthetics further supports the idea that oxidative stress serves as a core driver of anesthesia‐related cognitive impairment, and targeted antioxidant strategies effectively alleviate isoflurane‐triggered brain dysfunction by regulating the ACE2/Ang‐(1–7)/Mas signaling axis [40]. However, these intracellular pathways cannot completely explain the long‐lasting consequences of brief neonatal anesthetic exposure. Notably, the extracellular microenvironment, particularly PNNs, remains an underappreciated regulatory layer in anesthetic neurotoxicity [20]. Several preclinical studies have linked anesthetic exposure to PNN remodeling across developmental stages, with microglial phagocytosis being a well‐documented effector mechanism. In neonatal mice, repeated Sev administration induces microglia‐mediated PNN loss around prefrontal parvalbumin‐positive interneurons, driving social memory deficits [41]. In adult animals, ketamine anesthesia triggers microglia‐dependent breakdown of cortical PNNs to reopen critical period‐like plasticity [42]. Collectively, these findings suggest that myeloid cells are central mediators of anesthetic‐induced matrix remodeling. Despite these advances, critical mechanistic gaps remain in the literature. PNNs and BDNF/TrkB signaling maintain a reciprocal homeostatic relationship in the developing hippocampus: intact PNN architecture restricts excessive BDNF diffusion and stabilizes synaptic TrkB activity, while sustained BDNF/TrkB signaling further promotes the expression and assembly of core PNN components, such as Hapln1, to maintain ECM condensation [43, 44]. This bidirectional crosstalk is essential for the maturation and stabilization of developing neural circuits. However, its disruption following a neonatal anesthetic insult has not been systematically explored. The present study fills this critical knowledge gap and provides original evidence that CA2 regional PNN breakdown (specifically surrounding pyramidal neurons) and subsequent BDNF/TrkB signaling collapse are the central pathological events driving developmental Sev neurotoxicity.

To address the longstanding technical limitations in this field, we adopted spatial transcriptomics as the entry point for unbiased screening. Conventional bulk sequencing cannot resolve gene expression differences across discrete hippocampal subfields. Signals from small, selectively vulnerable regions are inevitably diluted by abundant tissues from adjacent areas. Single‐cell RNA‐seq achieves cellular resolution but loses its native anatomical context, making it difficult to map molecular alterations directly to intact cytoarchitectonic domains. By preserving native tissue architecture and mapping transcriptomic changes to their in situ locations, spatial transcriptomics allowed us to identify region‐specific ECM and PNN remodeling that would otherwise be masked in standard sequencing preparations. Building on these spatial findings, ultrastructural observation, epigenetic profiling, and multi‐dimensional behavioral assessment, we uncovered a novel and disease‐relevant H3K27me3–MMP9–PNN regulatory axis. Unlike acute intracellular stress responses, transient sevoflurane exposure induces stable epigenetic alteration, resulting in sustained MMP9 overactivation and region‐specific PNN erosion confined to the hippocampal CA2. This region‐selective vulnerability is closely associated with the unique molecular characteristics and delicate microenvironmental homeostasis of CA2 excitatory pyramidal neurons, which partially explains the specific social memory deficits observed in our behavioral tests [45]. To test the causal role of H3K27me3 loss in driving MMP9 upregulation and PNN degradation, we performed functional validation in primary cultured hippocampal neurons using GSK‐J4, a selective inhibitor of H3K27me3 demethylases. Preserving H3K27me3 levels via GSK‐J4 pretreatment significantly attenuated Sev‐induced MMP9 protein upregulation and mitigated the breakdown of core PNN structural components. These data provide direct functional evidence that reduced H3K27me3 occupancy at the *Mmp9* promoter acts as an upstream trigger of excessive MMP9 proteolytic activity and subsequent PNN disruption.

Accordingly, pharmacological inhibition of MMP9 by SB‐3CT effectively preserved PNN ultrastructure, restored downstream BDNF/TrkB signaling, and rescued synaptic and cognitive impairments. These intervention data consolidate the causal relationship between MMP9 hyperactivation, PNN degradation, and anesthetic neurotoxicity, and further identify ECM proteolysis as a viable and translatable therapeutic target for protecting the immature brain.

A major conceptual advancement of this study lies in the characterization of the bidirectional interaction between PNN integrity and BDNF/TrkB signaling in hippocampal CA2 pyramidal neurons. Under physiological conditions, PNNs function as physical and molecular barriers to confine the diffusion of neurotrophic factors, concentrate BDNF within synaptic microregions, and ensure the efficient binding and activation of TrkB receptors [46]. While previous work has shown that neonatal Sev exposure can trigger PNN erosion surrounding parvalbumin‐positive (PV^+^) interneurons and link this to social memory impairment [20], our findings extend this by identifying CA2 pyramidal neuron‐associated PNNs as a distinct and critical target of anesthetic neurotoxicity. Mechanistically, PNN‐derived chondroitin sulfate proteoglycans (CSPGs) restrict TrkB activation via the CSPG receptor protein tyrosine phosphatase sigma (PTPσ); enzymatic PNN degradation (e.g., ChABC treatment) potently elevates TrkB phosphorylation and signaling to reactivate neural plasticity [47]. Notably, this plasticity‐promoting effect is predominantly observed following acute, targeted enzymatic digestion of PNNs in the adult brain, with most reports focusing on PV‐positive interneurons in the visual cortex [48]. In this paradigm, ChABC selectively cleaves chondroitin sulfate side chains, leaving the core PNN protein scaffold largely intact. This targeted modification lifts PTPσ‐dependent inhibitory gating of TrkB without dismantling the broader perisynaptic architecture, a well‐characterized physiological mechanism that reinstates critical period‐like plasticity [44, 47]. In contrast, in pathological settings, sustained and widespread breakdown of core PNN structural proteins, compounded by region‐ and cell‐type‐specific differences in the PNN molecular makeup, drives fundamentally divergent effects on BDNF/TrkB signaling. The apparent paradox between the canonical barrier function of PNNs and our observed reduction in BDNF/TrkB signaling can be reconciled by distinguishing two fundamentally different modes of PNN disruption that operate over distinct time courses. Acute, selective cleavage of chondroitin sulfate side chains, similar to that with brief ChABC treatment, transiently relieves PTPσ‐mediated inhibitory gating of TrkB and increases local BDNF mobility, thereby enhancing synaptic plasticity [49, 50]. In contrast, sustained MMP9‐driven proteolysis triggered by neonatal Sev exposure targets the core structural backbone of PNNs, including aggrecan and Hapln1, rather than being restricted to glycosaminoglycan side chains alone [51]. Over the developmental timeframe examined in this study, progressive breakdown of this core scaffold does more than remove the diffusion barrier; it erodes the extracellular framework that anchors and clusters TrkB receptors at perisynaptic sites and destabilizes the dendritic spines and postsynaptic compartments that support the assembly of BDNF signaling complexes [52]. Consistent with the tight structural–functional coupling of synapses during developmental maturation, the progressive loss of intact synaptic substrates diminishes local BDNF production and impairs TrkB receptor membrane retention. The net effect is a sustained decline in BDNF/TrkB pathway signaling that outweighs any transient, early increase in BDNF bioavailability [53].

An impaired PNN structure disrupts this spatial restriction, disperses local BDNF, and reduces synaptic TrkB abundance, thereby weakening the core signaling cascade required for dendritic maintenance and synaptic plasticity [54]. To directly test whether diminished BDNF/TrkB signaling acts as a causal mediator of the observed synaptic deficits rather than a passive correlate, we performed rescue experiments in primary cultured hippocampal neurons via exogenous BDNF supplementation. Restoring BDNF availability significantly attenuated Sev‐induced downregulation of the presynaptic marker Synaptophysin and the postsynaptic scaffold protein PSD95. These data provide cellular‐level functional evidence that reduced BDNF/TrkB signaling actively drives synaptic structural damage, and confirm that synaptic impairments triggered by PNN erosion are mediated, at least in part, by disrupted BDNF/TrkB signaling. We acknowledge that these in vitro findings do not fully recapitulate the in vivo circuit and behavioral context; therefore, targeted in vivo modulation of the BDNF/TrkB pathway will be an important next step to extend these cellular observations to the systems level and behavioral phenotypes. Building on this downstream causal evidence, we sought to determine whether restoring the PNN architecture upstream could similarly rescue BDNF/TrkB signaling and synaptic integrity. We selected Hapln1 as our primary target for PNN reconstruction, due to its unique structural role in PNN assembly. Structurally, Hapln1 is a prototypical link protein in the central nervous system ECM that mediates high‐affinity cross‐linking between hyaluronan backbones and aggrecan monomers to form the basic repeating unit of the three‐dimensional PNN lattice [55]. Unlike core chondroitin sulfate proteoglycans such as aggrecan and versican, which are the bulk building blocks of the matrix, Hapln1 acts as a rate‐limiting determinant of overall network stability [56]. Even with an unaltered core proteoglycan abundance, partial Hapln1 loss destabilizes the supramolecular PNN scaffold and, rendering it more susceptible to proteolytic cleavage by MMP [56, 57]. Our gain‐ and loss‐of‐function experiments further verified this closed‐loop regulation: targeted Hapln1 overexpression efficiently reconstructed PNN architecture, stabilized local BDNF/TrkB signaling, and alleviated synaptic damage; whereas ChABC‐mediated enzymatic digestion of PNNs directly recapitulated BDNF/TrkB deficiency and cognitive dysfunction. These results demonstrate that the PNN–BDNF/TrkB homeostatic loop is indispensable for maintaining CA2 circuit stability and that its disruption is a key driving force of chronic neurodevelopmental impairments.

Compared to previous mechanistic studies in the field, our work shifts the research paradigm from single intracellular damage to an ECM‐centered regulatory network. Most anesthesia‐related studies emphasize acute neuronal injury [58], whereas our findings highlight that transient early‐life stress can reshape epigenetic modification and induce persistent ECM remodeling. This long‐term microenvironmental alteration provides a reasonable mechanistic interpretation of the delayed and progressive neurodevelopmental deficits after short‐term anesthetic exposure. Meanwhile, the identification of the H3K27me3–MMP9–PNN–BDNF/TrkB axis bridges epigenetic regulation, extracellular microenvironment remodeling, and neurotrophic signaling—offering a more comprehensive and integrated understanding of developmental brain injury that was lacking in prior work.

This study has several limitations. First, while our GSK‐J4 pharmacological inhibition experiments support a causal role for H3K27me3 reduction in Mmp9 upregulation, GSK‐J4 globally inhibits H3K27me3 demethylases and cannot distinguish effects specific to the Mmp9 promoter from broader epigenetic changes. Future studies using locus‐specific epigenetic editing (e.g., dCas9 fused to KDM6B or KMT6) at the Mmp9 promoter would provide more definitive evidence of direct transcriptional regulation. Second, despite the high selectivity of SB‐3CT for MMP9, the broad substrate spectrum of MMP9 means that further verification is required to confirm whether PNN structural proteins are the primary functional substrates mediating BDNF/TrkB dysfunction. Third, bulk tissue sequencing cannot distinguish cell‐type‐specific epigenetic changes in CA2 excitatory pyramidal neurons; future single‐cell multi‐omics analyses are necessary to refine this regional and cell‐specific mechanism. Fourth, the 3‐consecutive‐day Sev exposure paradigm used in this study is a well‐established preclinical model to induce robust and reproducible developmental neurotoxicity phenotypes for mechanistic dissection, but it does not fully recapitulate typical clinical exposure patterns in human neonates, who generally undergo less frequent anesthetic exposure for necessary surgical procedures. Thus, the translational relevance of our findings to clinical pediatric anesthesia settings requires cautious interpretation. Further validation in more clinically realistic exposure models and human translational studies is warranted.

To summarize, this study establishes an innovative ECM‐centric mechanism underlying Sev‐induced developmental neurotoxicity. Neonatal Sev exposure reduced H3K27me3‐mediated transcriptional repression, induced persistent MMP9 overexpression, and triggered CA2‐specific PNN degradation in excitatory pyramidal neurons. The collapse of PNN homeostasis disrupts the reciprocal PNN–BDNF/TrkB regulatory loop, leading to irreversible synaptic structural degeneration and functional failure, ultimately resulting in long‐term cognitive impairment. Both pharmacological MMP9 inhibition and genetic Hapln1‐based PNN reconstruction exert prominent neuroprotective effects. Beyond anesthetic neurotoxicity, this study provides a generalized mechanistic framework for understanding how early‐life environmental insults remodel the brain's extracellular microenvironment and program neurodevelopmental vulnerability. These findings deepen our mechanistic understanding of developmental brain injury and provide a preclinical rationale for exploring ECM‐targeted approaches as potential avenues for future studies on pediatric neuroprotection.

## 4 Conclusions

This study demonstrates that neonatal Sev exposure impairs the PNN surrounding the hippocampal CA2 pyramidal neurons via the H3K27me3–MMP9 cascade. PNN disruption interrupts the normal interplay between PNNs and BDNF/TrkB signaling, resulting in persistent synaptic impairment and cognitive deficits. Inhibiting MMP9 activity or increasing Hapln1 expression effectively preserves PNN structure and restores neurotrophic function, thereby mitigating neurodevelopmental deficits induced by early anesthetic exposure. Collectively, this signaling cascade is central to Sev‐induced developmental neurotoxicity and offers a viable therapeutic target for protecting the immature brain.

## 5 Materials and Methods

### 5.1 Animals

All C57BL/6J mice were obtained from the Animal Center of the Air Force Medical University (Xi'an, China). Camk2‐Cre mice were purchased from Cyagen Biosciences (Suzhou, China). GAD67‐GFP transgenic mice were obtained from The Jackson Laboratory (USA). All experimental procedures were approved by the Animal Care and Use Committee of Air Force Medical University (approval No. kq‐2021‐050), and complied with the Policies on the Use of Animals and Humans in Neuroscience Research (revised and approved by the Society for Neuroscience in 1995).

### 5.2 Experimental Design and Mice Treatments

Neonatal C57BL/6J mice at postnatal day 6 (P6) were randomly divided into four groups and subjected to 3 consecutive days of intervention, with daily 2 h exposure to 40% oxygen balanced with nitrogen: (1) Control (Con) group: intraperitoneal (i.p.) injection of DMSO as vehicle control; (2) Sev group: 3% sevoflurane (Sev, Cat# H20110142, USA) anesthesia; (3) SB‐3CT group: i.p. administration of 25 mg/kg SB‐3CT (MCE, HY‐12354) [37]; (4) SB‐3CT + Sev group: SB‐3CT injection 0.5 h prior to each Sev anesthesia session. For Hapln1 overexpression assays, postnatal day 6 (P6) Camk2‐Cre neonates were randomly assigned to four groups with the same 3‐day 40% O_2_ exposure regimen, and stereotaxic virus injection into the hippocampal CA2 region was performed at 1 month of age: (1) Dio‐EGFP group: Dio‐EGFP control virus injection; (2) Dio‐EGFP + Sev group: 3% Sev anesthesia plus Dio‐EGFP virus injection; (3) Dio‐Hapln1 group: Dio‐Hapln1 overexpression virus injection; (4) Dio‐Hapln1 + Sev group: 3% Sev anesthesia plus Dio‐Hapln1 virus injection.

The concentrations of Sev and oxygen were monitored continuously during anesthesia administration. During Sev exposure, the body temperature of mice was maintained with a warming blanket to prevent hypothermia. All animals were housed under standard laboratory conditions with ad libitum access to a regular chow diet and tap water. The animal facility was maintained at a constant temperature of 20°C–23°C with a 12 h light/dark cycle. Behavioral testing began on postnatal day 60, with all assays performed during the light phase of the circadian cycle (09:00–15:00). Mice were acclimated to the testing room for 30 min prior to each test. The behavioral battery was administered in order of increasing stress intensity: starting with novel object recognition, followed by social memory and contextual fear conditioning, and concluding with the Morris water maze. A 48 h recovery period separated consecutive assays to minimize carryover stress effects. Upon completion of all behavioral tasks, mice were euthanized for downstream histological and biochemical analyses.

### 5.3 Immunohistochemistry

Mice were intracardially perfused with 4% paraformaldehyde in phosphate buffer (Solarbio Life Science, China). For quantitative analysis, coronal sections spanning the full rostrocaudal extent of the hippocampus were sampled at 100 µm intervals (5–6 sections per mouse), with 3–6 mice included per experimental group. Serial coronal brain sections were blocked in PBS containing 3% BSA and 0.3% Triton X‐100, then incubated overnight at room temperature with the following primary antibodies: biotinylated Wisteria floribunda agglutinin (WFA; Vector Laboratories, 1:300), rabbit anti‐Aggrecan (Merck‐Millipore, MABT83, 1:200), rabbit anti‐NeuN (Merck Millipore, ABN78, 1:600), rabbit anti‐GFAP (GeneTex, GTX108711, 1:200), mouse anti‐CC1 (Sigma–Aldrich, OP80, 1:100), rabbit anti‐Iba1 (WAKO, Lek0542, 1:200), rabbit anti‐Camk2 (GeneTex, GTX135117, 1:200), mouse anti‐CaMKIIα (Abcam, ab22609, 1:200), goat anti‐BDNF (Vector Laboratories, EB08117, 1:500), rabbit anti‐VGlut2 (Abcam, ab216463, 1:200), chicken anti‐MAP2 (Abcam, ab5392, 1:500), and rabbit anti‐MMP9 (Abclonal, A25299, 1:200).After PBS washes, sections were incubated for 3 h at room temperature in the dark with corresponding Alexa Fluor 488‐ or 594‐conjugated secondary antibodies (donkey anti‐rabbit, donkey anti‐mouse, donkey anti‐chicken, and donkey anti‐goat; Jackson ImmunoResearch, 1:500) or Alexa Fluor 488‐conjugated streptavidin (Cell Signaling Technology, 89067S, 1:500). Sections were then counterstained with DAPI (Beyotime, 1:1000) or Hoechst (Sigma, 1:1000) for 10 min, and images were acquired using a confocal microscope.

### 5.4 Western‐Blotting

Hippocampal tissue samples were homogenized in RIPA lysis buffer supplemented with a protease inhibitor cocktail. Protein concentration was determined via the BCA assay. Protein samples were resolved by 8%–15% sodium dodecyl sulfate‐polyacrylamide gel electrophoresis (SDS‐PAGE) and subsequently transferred onto polyvinylidene fluoride (PVDF) membranes. The membranes were blocked with Tris buffered saline (TBS) containing 5% nonfat milk and 0.1% Tween 20 for 1h at room temperature, followed by an overnight incubation at 4°C with primary antibodies, including: rabbit anti‐hapln1 (proteintech, 31646‐1‐AP, 1:1000), rabbit anti‐Aggrecan (Merck‐Millipore, MABT83, 1:1000), rabbit anti‐PSD‐95 (Abcam, ab76115, 1:1000), rabbit anti‐PSD‐95 (proteintech, 20665‐1‐AP, 1:1000), rabbit anti‐Synaptophysin (Abcam, ab32127, 1:1000), rabbit anti‐Synaptophysin (proteintech, 17785‐1‐AP, 1:1000), rabbit anti‐BDNF (proteintech, 28205‐1‐AP, 1:1000), rabbit anti‐TrkB (proteintech, 13129‐1‐AP, 1:1000), rabbit anti‐H3K27me3 (Abclonal, A22006, 1:1000), rabbit anti‐H3 (Cell Signaling Technology, #9715, 1:1000), rabbit anti‐MMP9 (Abclonal, A25299, 1:1000), rabbit anti‐β‐Actin (proteintech, 20536‐1‐AP, 1:4000). After washing with TBST, the membranes were incubated with horseradish peroxidase (HRP)‐conjugated anti‐rabbit and HRP‐conjugated anti‐mouse secondary antibodies (1:5000; Proteintech, Cat#:SA00001‐2, Cat#:SA00001‐1) for 1h at room temperature. Protein bands were visualized using an enhanced chemiluminescence (ECL) kit (Thermo, Cat#:32106). Images were analyzed by ImageJ.

### 5.5 Primary Neuron Culture and Treatment

Hippocampal neurons were isolated from E15‐ 17 mice. Briefly, a cesarean section was performed, and the embryos were taken out carefully. The brains were dissected and put into a dish containing D‐ hanks buffer. We carefully dissected out the hippocampus, removed the meninges, and cut the tissue into size of 1 mm3 and digested the tissue with 0.125% trypsin for 10 min. After trituration, cells were centrifuged for at 1000 rpm for 5 min and then cultured in serum‐ free B27/neurobasal medium. For sevoflurane exposure, sevoflurane was delivered from an anesthesia machine to a sealed plastic box in a 37°C incubator. An anesthetic gas monitor (Drager) was used to continuously monitor the concentrations of carbon dioxide, oxygen, and sevoflurane. At 2 days in vitro (2 DIV), primary hippocampal neurons were exposed to 3% sevoflurane (21% O_2_ and 5% CO_2_) for 2 h per day across 3 consecutive days. At 14 days in vitro, neurons were collected for experiments.

GSK‐J4 (MCE, HY‐15648B) was dissolved in dimethyl sulfoxide (DMSO) to a stock concentration of 10 mM and stored at −20°C. Primary hippocampal neurons at DIV 2 were pretreated with GSK‐J4 at a final concentration of 0.5 µM [59]for 1 h prior to sevoflurane exposure. Control cultures received an equal volume of DMSO vehicle. Following sevoflurane exposure, neurons were maintained in culture medium containing GSK‐J4 or vehicle until harvest for downstream analysis.

Recombinant human BDNF (MCE, HY‐P7116) was reconstituted in sterile PBS containing 0.1% BSA to a stock concentration of 10 µg/mL and stored at −80°C in single‐use aliquots. Within 30 min after sevoflurane exposure, primary hippocampal neurons were treated with BDNF at a final concentration of 10 ng/mL [60]. Control cultures received an equal volume of PBS/0.1% BSA vehicle until harvest for downstream analysis.

### 5.6 Golgi Staining

Mice were perfused with PBS at 8 weeks of age. Brains were rapidly dissected and immersed in Golgi staining solution, which contained 5% potassium dichromate (MP 021563389), 5% mercuric chloride (Sigma–Aldrich M1136), and 5% potassium chromate (Sigma–Aldrich 529508), followed by light‐protected incubation for 7 days. For Golgi staining processing, brain sections were rinsed with distilled water, dehydrated through a graded ethanol, and then treated with ammonia (3:1). The sections were subsequently washed and incubated in 5% sodium thiosulfate for 10 min and then dehydrated with degraded ethanol and clarified with xylene. In the end, the sections were observed under bright field of Olympus FV3000. The images were taken by z‐stack scanning with an excitation wavelength of 405 nm, and the virtual color was converted into green color. For dendrite branching, Sholl analysis was conducted. For spine density, dendrites of pyramidal neurons in the CA region were reconstructed and analyzed by using IMARIS.

### 5.7 Electron Microscopic Study

#### 5.7.1 Transmission Electron Microscopy (TEM) for Postsynaptic Density (PSD) Analysis

Animals were transcardially perfused with a fixative solution containing 4% paraformaldehyde and 1% glutaraldehyde. Hippocampal tissues were sectioned into 50 µm slices using a vibratome, post‐fixed with 1% osmium tetroxide, dehydrated through a graded ethanol series, infiltrated with propylene oxide, and flat‐embedded in Epon 812 resin. Ultrathin sections (70–90 nm) were cut using an ultramicrotome, counterstained with uranyl acetate and lead citrate, and examined under a JEM‐1230 transmission electron microscope. PSD thickness was manually measured using electron micrographs.

#### 5.7.2 Immunoelectron Microscopy (IEM) for WFA‐Labeled PNN Localization

For WFA immunogold staining, ultrathin sections were blocked with 5% bovine serum albumin (BSA) in PBS for 30 min at room temperature to eliminate nonspecific binding, followed by overnight incubation at 4°C with biotinylated Wisteria floribunda agglutinin (WFA; Vector Laboratories, 1:150 dilution) diluted in PBS containing 1% BSA. After extensive PBS washes, sections were incubated with streptavidin‐conjugated colloidal gold (10–15 nm, 1:50 dilution) for 2 h at room temperature in the dark. Sections were counterstained with uranyl acetate and lead citrate, and imaged using the JEM‐1230 electron microscope for ultrastructural localization of WFA‐positive perineuronal nets (PNNs).

### 5.8 H3K27me3 Chromatin Immunoprecipitation (ChIP) Sequencing and ChIP‐qPCR Analysis

Profiling of H3K27me3 via ChIP‐seq was conducted in mouse hippocampal tissue following standard protocols described elsewhere. Tissues were washed twice with ice–cold PBS containing protease inhibitors, then homogenized and lysed in SDS lysis buffer supplemented with phosphatase and protease inhibitor cocktails. Chromatin was fragmented to 200–500 bp via sonication (Bioruptor) on ice, with fragmentation efficiency verified via agarose gel electrophoresis. The sonicated chromatin supernatants were diluted with ChIP dilution buffer and pre‐cleared with protein A/G magnetic beads for 1 h at 4°C to reduce nonspecific binding. For each sample, equal aliquots of chromatin were incubated overnight at 4°C with H3K27me3‐specific antibody or corresponding IgG (negative control). Antibody‐chromatin complexes were captured by protein A/G magnetic beads, followed by sequential washing with low‐salt buffer, high‐salt buffer, and TE buffer. Library construction and high‐throughput PE150 sequencing were performed by Kangce Technology (Wuhan, China) on an Illumina sequencing platform, and library quality was assessed by Kangce Technology using an Agilent 2100 Bioanalyzer prior to sequencing.

Eluted DNA from ChIP samples was analysed by SYBR Green‐based quantitative real‐time PCR. Primers were designed to amplify the endogenous Mmp9 promoter (forward: 5′‐TTGTTAGCTGCCATGTGGGT; reverse: 5′‐CCTTTGGGGGCTGAAGACAT). Input DNA was used for normalisation, and IgG immunoprecipitates served as a negative control to subtract nonspecific background binding. Relative H3K27me3 enrichment at the Mmp9 promoter was calculated with the 2^(−^ΔΔCt^) method. All reactions were run in technical triplicate to guarantee reproducibility across independent tests.

### 5.9 Patch‐Clamp Recordings

Whole‐cell patch‐clamp recordings were performed on cultured hippocampal neurons. A gigaseal was first formed between the recording pipette and neuronal membrane, followed by gentle suction to rupture the membrane patch and establish the whole‐cell configuration. After a 3 min stabilization interval, excitatory postsynaptic currents (EPSCs) were recorded in voltage‐clamp mode at a holding potential of −70 mV to establish a stable baseline.

### 5.10 Novel Object Recognition Test

Day 1, mice were habituated to an empty open‐field arena (40 × 40 × 35 cm) for 10 min. Day 2, mice were allowed to freely explore two identical objects placed in the open field for 10 min. One hour later, one of the familiar objects was replaced with a novel object, then the mice were reexposed to the arena for a 5 min memory test. Behavioral performance was recorded using video tracking software, and we calculated the ratio of time spent exploring the novel relative to the familiar object.

### 5.11 Two‐Trial Social Memory Test

Social memory was assessed in a standardized two‐trial social recognition paradigm, as outlined in an earlier report [61]. The subject mice were 2 months old. Before testing, the mice were individually housed in a clean polycarbonate cage for 30 min. Following habituation, a same‐sex C57BL/6J stimulus mouse (6 weeks old) was introduced into the subject mouse's cage, and social interaction was monitored for 2 min (Trial 1). The lid of the cage was removed to allow filming of social activities. The stimulus mouse was then removed and returned to its home cage, and the total duration of social interaction was recorded. After a 30 min intertrial interval, another stimulus mouse was reintroduced into the subject mouse's cage for a second 2 min interaction session (Trial 2), and social interaction time was recorded as described for Trial 1.

### 5.12 Fear Conditioned Memory

All mice were pre‐exposed to the startle chambers (San Diego Instruments) 3 days before training. During cued fear training, mice received five paired trials of a conditioned stimulus (CS; a 30 s tone, 6 or 12 kHz, 90 dB) and an unconditioned stimulus (US; a 500 ms, 1.0 mA foot shock), with a 5 min intertrial interval. Startle responses to the shocks and the percentage of freezing time evoked by the tones were measured using Xmaze software (XinRuan Informatics Co.).

### 5.13 Morris Water Maze Test

The water maze consisted of a circular container (120 cm in diameter, 40 cm in height). The water maze was divided into four quadrants. The escape platform was submerged 1.5 cm below the water surface. During training, the mice were randomly placed into the water, facing the wall, from one of the four quadrants. The time taken to find the platform was recorded as the escape latency. Mice that failed to locate the platform within 60s were guided to the platform to facilitate learning. Each mouse completed four training trials per day for four consecutive days. On day 5, a probe trial was conducted after the platform was removed, allowing mice to swim freely for 60 s. The total time spent in the target quadrant, where the platform had previously been positioned, was measured to evaluate reference memory. Swimming paths and escape behaviors were recorded and analyzed using SMART v.3.0.

### 5.14 Stereotaxic Surgery

All surgeries were performed under aseptic conditions, and body temperature was maintained using a heating blanket throughout the procedure. Standard protocols were followed for stereotaxic injection. A total of 200 nL of viral suspension was bilaterally injected into the hippocampal CA2/3 regions of Camk2‐Cre mice. The two viral constructs used were pAAV‐EF1a‐DIO‐EGFP (catalog no. PT‐0795) and pAAV‐EF1a‐DIO‐Hapln1‐EGFP (catalog no. PT‐8069), both packaged in the AAV2/9 serotype at a titer of 5.18 × 10^1^ ^2^ vg/mL (BrainVTA, Wuhan, China) [62]. The stereotaxic coordinates relative to bregma were as follows: anteroposterior, −1.6 mm; mediolateral, ± 1.6 mm; dorsoventral, −1.7 mm [63]. Virus was infused at a constant rate of 50 nL/min using a 10 µL microsyringe (Sangon, Shanghai, China) driven by a microsyringe pump (RWD, Shenzhen, China). To prevent fluid reflux, the injection needle remained in place for 10 min before being slowly withdrawn. The scalp was closed with three to four sutures after injection. Mice were allowed to recover for three weeks to permit sufficient viral expression before behavioral testing. For ChABC experiments, 1 µL of ChABC or vehicle PBS (25 U/mL) was bilaterally delivered into the hippocampal CA2 region [63]. One week after surgery, injected mice were either euthanized for immunohistochemical analysis or subjected to behavioral assessments.

### 5.15 Statistical Analysis

Statistical analyses were performed with GraphPad Prism version 10.5 (GraphPad Software Inc., San Diego, CA, USA). All behavioral measurements and data analyses were performed by an investigator blinded to the experimental grouping. Each behavior test was conducted using distinct groups of animals. Data normality was assessed using the Shapiro–Wilk test. Two‐group comparisons were performed with a two‐tailed unpaired Student's *t*‐test, while one‐way ANOVA followed by Tukey's post hoc test was applied for multi‐group comparisons. For the Morris water maze escape latency during the training phase, where the same animals were tested repeatedly across days, two‐way repeated‐measures ANOVA followed by Sidak's post hoc test was used [64]. All data were presented as the mean ± standard error (SEM). All experiments were independently repeated at least three times (n ≥ 3 biological replicates). Detailed sample sizes and specific statistical methods for each analysis are specified in the corresponding figure legends. A *p*‐value < 0.05 was considered statistically significant.

## Author Contributions

Behavior analysis: L. L., J. W., T. W.; immunohistochemistry: Y. L., Y. Z, L. L.; western‐blotting: J. W., T. W., P. G.; Data analysis: Y. L., Y. Z, P. G., G. H., R. C.; morphological study: L. L., J. W., G. H., Y. F.; manuscript preparation: L. L., H. Z., H. Z., S. W.; experimental design: L. L., H. Z., S. W., H. Z.; financial support: H. Z., L. L., H. Z. All authors have reviewed and approved the final version of the manuscript.

## Ethics Statement

All experimental procedures were carried out according to the protocols approved by the Animal Care and Use Committee of the Fourth Military Medical University (FMMU; Approval No. kq‐2021‐050).

## Conflicts of Interest

The authors declare no conflicts of interest.

## Supporting information




**Supporting File**: advs78006‐sup‐0001‐SuppMat.docx.

## Data Availability

Raw sequencing data generated in this study have been deposited in the Genome Sequence Archive (GSA; accession number: CRA047885). All remaining data supporting the findings of this study are available from the corresponding author upon reasonable request.
