# Supplementary material for: Neonatal Sevoflurane Exposure Induces Long‐Term Cognitive Impairment via Epigenetically Mediated MMP9 Activation and Perineuronal Net Disruption in the Hippocampal CA2
Source: Adv Sci (Weinh). 2026 Sep 29:e78006. Online ahead of print. doi: 10.1002/advs.78006 (PMC13624421; doi:10.1002/advs.78006)
Supplement: Supplementary file 1 [file ADVS-9999-e78006-s001.docx]

**Title:** Neonatal Sevoflurane Exposure Induces Long-Term Cognitive Impairment via Epigenetically Mediated MMP9 Activation and Perineuronal Net Disruption in the Hippocampal CA2

**Running title**: Sevoflurane Disrupts Hippocampal CA2 Perineuronal Nets via the H3K27me3-MMP9 Axis to Impair Cognitive Development

**Authors:**

Lirong Liang^1#*^, Jiachen Wang^1#^, Taozhi Wang^2,4#^, Yanling Li^1^, Youyi Zhao^1^, Guanghui Hao^1^, Peiqin Gong^1^, Ruling Chen^1^, Yilin Fang^1^, Shengxi Wu^3^*, Haopeng Zhang^1^*, Hui Zhang^1^*

**Author information:**

*^1^State Key Laboratory of Oral & Maxillofacial Reconstruction and Regeneration, National Clinical Research Center for Oral Diseases, Shaanxi Engineering Research Center for Dental Materials and Advanced Manufacture, Department of Anesthesiology, School of Stomatology, Fourth Military Medical University, Xi'an, Shaanxi 710032, China*

*^2^Department of Anesthesiology, Zhujiang Hospital, Southern Medical University, Guangzhou, Guangdong 510280, China*

*^3^Department of Neurobiology and Institute of Neurosciences, School of Basic Medicine, Fourth Military Medical University, Xi’an, Shaanxi 710032, P. R. China*

*^4^Department of Anesthesiology and Perioperative Medicine, Xijing Hospital, Fourth Military Medical University, Xi’an 710032, China*

^#^ These authors contribute equally to the work.

**^*^Corresponding authors:**

Dr. Hui Zhang, State Key Laboratory of Oral & Maxillofacial Reconstruction and Regeneration, National Clinical Research Center for Oral Diseases, Shaanxi Engineering Research Center for Dental Materials and Advanced Manufacture, Department of Anesthesiology, School of Stomatology, Fourth Military Medical University, Xi’an, Shaanxi 710032, P. R. China. Tel: +86-29-84776115, email: [fuming@fmmu.edu.cn](mailto:fuming@fmmu.edu.cn); [zhanghuifmmua@163.com](mailto:zhanghuifmmua@163.com)

Dr. Haopeng Zhang, State Key Laboratory of Oral & Maxillofacial Reconstruction and Regeneration, National Clinical Research Center for Oral Diseases, Shaanxi Engineering Research Center for Dental Materials and Advanced Manufacture, Department of Anesthesiology, School of Stomatology, Fourth Military Medical University, Xi'an, Shaanxi 710032, P. R. China. Tel: +86-29-84776115, email: haopeng.zhang@foxmail.com

Dr. Shengxi Wu, Department of Neurobiology and Institute of Neurosciences, School of Basic Medicine, Fourth Military Medical University, Xi’an, Shaanxi 710032, P. R. China. Tel:+86-29-8474557, email: shengxi@fmmu.edu.cn

Dr. Lirong Liang, State Key Laboratory of Oral & Maxillofacial Reconstruction and Regeneration, National Clinical Research Center for Oral Diseases, Shaanxi Engineering Research Center for Dental Materials and Advanced Manufacture, Department of Anesthesiology, School of Stomatology, Fourth Military Medical University, Xi'an, Shaanxi 710032, P. R. China. Tel: +86-29-84776119, email: [18991802552@163.com](mailto:18991802552@163.com)

**Supplementary Figure 1.** **Neonatal Sev exposure induces persistent cognitive and social memory deficits in adult mice.**

A) Schematic of the experimental timeline. Mice were exposed to 3% Sev or Con for 2 hours daily on postnatal days 6–8. B-D) Novel object recognition test. B) Schematic of the training (10 min) and testing (5 min, 1 h post-training) procedures. C) Exploration time of the novel object. D) Recognition index. E-F) Social memory test. E) Schematic of the social interaction paradigm. F) Interaction time with the novel stranger mouse. G-H) Fear conditioning test. G) Schematic of the fear conditioning protocol. H) Percentage of freezing time during the test. I–K) Morris water maze test. I) Representative swim path traces during the probe trial. J) Escape latency during the training phase. K) Time spent in the goal quadrant during the probe trial. Data are presented as mean ± SEM. n=6 mice per group. Statistical comparisons were performed using unpaired Student’s t-test (C, D, F, H, K) and two-way repeated-measures ANOVA followed by Sidak's post hoc test (J). **P* < 0.05, ***P* < 0.01.

Supplementary Figure 1

**Supplementary Figure 2. Layer annotation based on cytoarchitecture and known marker gene expression.**

A-D) For each of the four samples (Con1, Con2, Sev1, Sev2), three panels are shown: H&E staining of the coronal brain section, with the total number of sequencing spots indicated (left); spot-level graph-based clustering with anatomical brain regions demarcated by dashed red lines (middle); and UMAP projection of spot-level transcriptional signatures, color-coding consistently with the clustering legend (right).

Supplementary Figure 2

**Supplementary Figure 3. Efficient Decellularization of Mouse Brain Slices.**

A) Pre-treatment workflow: mouse brain slices were prepared, pre-treated with PBS, and the target brain region was dissected for subsequent decellularization. B) Decellularization procedure. Brain slices underwent 3 sequential cycles of treatment, each including dH₂O, 2% sodium deoxycholate (SDC), DNase I/NaCl, dH₂O, Triton X-100, and DNase I/NaCl steps. Representative images at each step across all 3 cycles are shown. C) DAPI staining to assess decellularization efficiency. Pre-decellularization control (PC) and slices after 3 decellularization cycles (3 Cycle) are shown. Scale bar = 50 μm. n=3 mice per group.

Supplementary Figure 3

**Supplementary Figure 4.** **Neonatal Sev Exposure Reduces PNN and Aggrecan Expression in the Mouse Hippocampus.**

A-C) WFA staining and quantification of WFA intensity in the CA1 (A), CA3 (B), and dentate gyrus (DG) (C) at 60 days post-exposure (dpe). Scale bars = 50 μm. D, E) Aggrecan immunofluorescence (D) and quantification (E) in the CA2 region at 7, 14, and 30 dpe in Con and Sev mice. Scale bar = 50 μm. F) Representative triple immunofluorescence staining for WFA (green), aggrecan (red), and Hoechst (nuclear marker, blue) in Con and Sev mice. Scale bar = 50 μm. G) Quantification of co-localized WFA and aggrecan intensity shown in F. Data are presented as mean ± SEM. n = 3 mice per group. Statistical comparisons were performed using unpaired Student’s t-test (A, B, C, E, G). ns, not significant; ****P* < 0.001.

Supplementary Figure 4

**Supplementary Figure 5.** **Sev exposure preferentially disrupts perineuronal nets around CA2 excitatory neurons.**

A-B) Co-immunofluorescence staining of CaMKIIα (green) and WFA (magenta) in the CA2 region of Con and Sev mice (A), and quantification of WFA fluorescence intensity surrounding CaMKIIα-positive neurons (B). Scale bar = 50 μm. C-D) Co-immunofluorescence staining of GAD67-GFP (green) and WFA (magenta) in the CA2 region of Con and Sev mice (C), and quantification of WFA fluorescence intensity surrounding GAD67-GFP-positive neurons (D). GAD67-positive inhibitory neurons were identified by GFP fluorescence in GAD67-GFP reporter mice. Scale bar = 50 μm.

Data are presented as mean ± SEM. n = 3 mice per group. Statistical comparisons were performed using unpaired Student's t-test. ∗*P* < 0.05; ns, not significant.

Supplementary Figure 5

**Supplementary Figure 6. ChABC-mediated degradation of PNNs in the mouse hippocampus.**

A) Schematic of the experimental timeline. Mice were exposed to Sev or Con at P6–P8, received stereotaxic intrahippocampal injection of ChABC or PBS at P55, and underwent immunofluorescence staining starting at P60. B) Representative immunofluorescence staining for WFA (green) and CA2 marker PCP4 (red) in hippocampal sections from Con + PBS or Con + ChABC mice. Scale bar = 100 μm. C) Split-channel and merged immunofluorescence images of PCP4 (red), WFA (green), Hoechst (blue) in the CA2 region of Con + PBS and Con + ChABC mice. Scale bar = 50 μm. D) Quantification of WFA^+^PCP4^+^ intensity in Con+PBS and Con + ChABC mice. Data are presented as mean ± SEM. n = 3 mice per group. Statistical comparisons between groups were performed using Student’s t-test. *****P* < 0.0001.

Supplementary Figure 6

**Supplementary Figure 7. Validation of rAAV-DIO-Hapln1 expression in the hippocampal CA2/3 region.**

A–D) Representative immunofluorescence images of the hippocampal CA2/3 region in Camk2-Cre mice after stereotaxic injection of rAAV-DIO-Hapln1-EGFP. A) Hoechst staining (blue) delineates the CA2 and CA3 subfields. B) Dio-Hapln1-EGFP expression (green) is detected in the pyramidal cell layers of both CA2 and CA3. C) WFA staining (magenta) labels perineuronal nets. D) Merged image showing colocalization of Dio-Hapln1-EGFP with WFA-positive PNNs. Dashed lines indicate the CA2/CA3 boundary. Scale bar = 50 μm.

Supplementary Figure 7

**Supplementary Figure 8. ChABC-mediated PNN degradation exacerbates Sev-induced cognitive deficits in adult mice.**
A) Schematic of the experimental timeline. Mice were exposed to Sev or Con at P6–P8, received stereotaxic intrahippocampal injection of ChABC or PBS at P55, and underwent behavioral tests starting at P60. B-C) Novel object recognition test: novel object exploration time (B) and recognition index (C) across four groups. D) Social memory test: interaction time across habituation and novel mouse trials across the four groups. E) Fear conditioning test: percentage of freezing time across the four groups. Data represent the mean ± SEM. n = 6 mice per group. Statistical comparisons were performed using One-way ANOVA followed by Tukey's post hoc test (B, C, D, E). **P*< 0.05, ***P* < 0.01, ****P* < 0.001, *****P* < 0.0001; ns, not significant.

Supplementary Figure 8

**Supplementary Figure 9. Sev exposure and ChABC-mediated degradation of PNNs impair PNN formation in cultured hippocampal neurons.**

A) Schematic of the experimental workflow. Hippocampal neurons were isolated from E15 mouse embryos and cultured for subsequent Sev exposure or ChABC treatment. B) Representative immunofluorescence staining for MAP2 (gray) and WFA (green) in Con and Sev-treated cultured neurons. Red triangles denote WFA-positive neurons. Scale bar = 20 μm. C) Quantification of WFA-positive neuron number. Sev-treated neurons showed a significant reduction in WFA^+^ neurons compared to Con. D) Time-course immunofluorescence staining for WFA (green), MAP2 (white), and DAPI (blue) in cultured neurons treated with PBS or ChABC for 2, 8, 12, or 48 h. Scale bar = 20 μm. E) Representative immunofluorescence staining for MAP2 (purple) and WFA (green) in PBS-treated (Con + PBS) or ChABC-treated neurons. Scale bar = 20 μm. F) Quantification of WFA-treated neuron number. Data are presented as mean ± SEM. n = 6 independent cultures per group. Statistical comparisons between groups were performed using unpaired Student's t-test (C, F). *****P* < 0.0001.

Supplementary Figure 9

**Supplementary Figure 10. ChABC-mediated enzymatic degradation of PNNs impairs synaptic integrity, dendritic morphology, and synaptic function**

A-B) Western blotting and quantification of Hapln1 protein levels in hippocampal tissues from Camk2-cre mice treated with Dio-EGFP, Dio-EGFP+Sev, Dio-Hapln1, Dio-Hapln1+Sev, ChABC, or PBS. C-D) Western blotting and quantification of PSD95 and synaptophysin in the hippocampal from ChABC and PBS-treated mice. E-H) Representative TEM images (E) and quantification of PSD number (F), length (G), and thickness (H) in the hippocampal of ChABC- and PBS-treated mice. Presynaptic terminals (yellow), postsynaptic terminals (green), and PSD (purple) are pseudocolored. Scale bar = 250 nm. I-K) Western blotting and quantification of BDNF and TrkB protein levels in the hippocampal of ChABC- and PBS-treated mic. L) Representative MAP2 staining (top) and Sholl analysis (bottom). Scale bar = 20 μm. M-O) Sholl analysis and quantification of dendritic intersections (M), dendrite number (N), and total dendrite length (O). P-R) Representative traces of sEPSC (P) and quantification of sEPSC frequency (Q) and amplitude (R) in PBS- and ChABC-treated neuron. Data are presented as mean ± SEM. n = 6 mice per group (A, B, C, D, I, J, K); n = 3 mice per group (E, F, G, H); n = 30–40 neurons per group for morphological analyses (L, M, N, O); n = 8–9 neurons per group for electrophysiology (Q, R). Statistical comparisons were performed using unpaired Student’s t-test (D、F、G、H、J、K、N、O、Q and R) or one-way ANOVA followed by Tukey's post hoc test (B, M). **P* < 0.05, ***P* < 0.01, ****P* < 0.001, *****P* < 0.0001.

Supplementary Figure 10

**Supplementary Figure 11. GSK-J4 and exogenous BDNF rescue sevoflurane-induced PNN and synaptic protein deficits in cultured hippocampal neurons.**
A-B) Representative immunofluorescence staining for WFA (green) and MAP2 (magenta) (A), and quantification of WFA-positive neuron number (B), across three groups: Con + Vehicle, Sev + Vehicle, and Sev + GSK-J4. Scale bar = 20 μm. C-D) Representative Western blots (C) and quantification (D) of MMP9 protein levels, normalized to β-actin, across the three groups. E-F) Representative Western blots (E) and quantification of PSD95 and synaptophysin protein levels, normalized to β-actin (F), across three groups: Con + Vehicle, Sev + Vehicle, and Sev + BDNF. Data are mean ± SEM. n = 6 independent cultures per group. Statistical comparisons were performed using one-way ANOVA followed by Tukey's post hoc test. ∗*P* < 0.05, ∗∗*P* < 0.01, ∗∗∗*P* < 0.001, ∗∗∗∗*P* < 0.0001.

Supplementary Figure 11
